# Ultrafast Metal‐Free Microsupercapacitor Arrays Directly Store Instantaneous High‐Voltage Electricity from Mechanical Energy Harvesters

**DOI:** 10.1002/advs.202400697

**Published:** 2024-03-19

**Authors:** Shiqian Chen, Zheng Li, Po‐Han Huang, Virginia Ruiz, Yingchun Su, Yujie Fu, Yolanda Alesanco, B. Gunnar Malm, Frank Niklaus, Jiantong Li

**Affiliations:** ^1^ KTH Royal Institute of Technology School of Electrical Engineering and Computer Science Division of Electronics and Embedded Systems Electrum 229 Kista 16440 Sweden; ^2^ KTH Royal Institute of Technology School of Electrical Engineering and Computer Science Division of Micro and Nanosystems Stockholm SE‐100 44 Sweden; ^3^ CIDETEC Basque Research and Technology Alliance (BRTA) Po. Miramón 196 Donostia‐San Sebastián 20014 Spain; ^4^ Present address: International Research Center in Critical Raw Materials‐ICCRAM Universidad de Burgos Plaza Misael Bañuelos s/n Burgos E‐09001 Spain

**Keywords:** droplet‐based electricity generators, full printing, microsupercapacitor arrays, on‐paper electronics, PEDOT:PSS

## Abstract

Harvesting renewable mechanical energy is envisioned as a promising and sustainable way for power generation. Many recent mechanical energy harvesters are able to produce instantaneous (pulsed) electricity with a high peak voltage of over 100 V. However, directly storing such irregular high‐voltage pulse electricity remains a great challenge. The use of extra power management components can boost storage efficiency but increase system complexity. Here utilizing the conducting polymer PEDOT:PSS, high‐rate metal‐free micro‐supercapacitor (MSC) arrays are successfully fabricated for direct high‐efficiency storage of high‐voltage pulse electricity. Within an area of 2.4 × 3.4 cm^2^ on various paper substrates, large‐scale MSC arrays (comprising up to 100 cells) can be printed to deliver a working voltage window of 160 V at an ultrahigh scan rate up to 30 V s^−1^. The ultrahigh rate capability enables the MSC arrays to quickly capture and efficiently store the high‐voltage (≈150 V) pulse electricity produced by a droplet‐based electricity generator at a high efficiency of 62%, significantly higher than that (<2%) of the batteries or capacitors demonstrated in the literature. Moreover, the compact and metal‐free features make these MSC arrays excellent candidates for sustainable high‐performance energy storage in self‐charging power systems.

## Graphical abstract

Advanced mechanical energy harvesters, such as droplet‐based electricity generators (DEG) produce high‐voltage (>100 V) pulse electricity, whereas existing energy storage devices can only directly store such electricity at an efficiency of <2%. Here, ultrafast metal‐free large‐scale (≈100 cells) micro‐supercapacitor arrays are fully printed on paper substrates to directly store DEG‐produced high‐voltage pulse electricity at efficiency >60%.

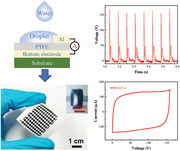

## 1 Introduction

The growing global awareness of energy crisis, climate change, and environmental pollution has stimulated extensive research, aimed at harvesting clean and renewable energy from ambient mechanical sources, such as wind, ocean waves, water droplets, and human motion.^[^ ^1^ ^]^ State‐of‐the‐art mechanical energy harvesters enable production of electricity with an instantaneous power density of up to 10 MW m^−2^ and output peak voltage at kV levels^[^ ^1^ ^]^ through various mechanisms, including droplet‐based electricity generators^[^ ^2^ ^]^ and triboelectric nanogenerators.^[^ ^1^, ^3^, ^4^ ^]^ Because ambient mechanical energy sources are usually intermittent, the output electricity of mechanical energy harvesters is not available all the time and typically features pulsed waveforms with random amplitudes and frequencies.^[^ ^5^ ^]^ Therefore, it is necessary to save the resulting irregular electricity into energy storage devices, such as batteries or supercapacitors, to form stable and sustainable power sources for electronics.^[^ ^5^, ^6^ ^]^ However, present energy storage devices only work at low voltage (<10 V) and low charging rate (<100 mV s^−1^).^[^ ^6^ ^]^ Their storage efficiency reduces drastically^[^ ^7^, ^8^, ^9^ ^]^ to below 2% when directly storing the electricity harvested from a mechanical energy source which typically is at high voltage (>100 V) yet at an instantaneous time scale of milliseconds.^[^ ^2^ ^]^ At present, additional electronic components, e.g., buck converters,^[^ ^5^, ^10^ ^]^ inductors,^[^ ^7^ ^]^ capacitors,^[^ ^11^ ^]^ and transformers,^[^ ^8^, ^12^ ^]^ are needed in the power management modules to step down the high voltage or buffer the electricity. Not only do these added components increase the complexity and form factor of the energy harvesting systems, but they also induce considerable extra losses during energy conversion. Thus, innovative energy storage devices are desired that possess high energy density, wide working voltage window (hereafter simplified as voltage window) of >100 V, and high‐rate capability to directly store the instantaneous high‐voltage electricity with high efficiency.

Recently, there has been a growing research interest in large‐scale micro‐supercapacitor (MSC) arrays.^[^ ^13^, ^14^, ^15^, ^16^, ^17^ ^]^ A series connection of hundreds of MSCs allows to both widen the voltage window^[^ ^16^ ^]^ over 100 V and increase the rate capability^[^ ^13^ ^]^ to over 1 V s^−1^. These merits provide a solution for directly storing the high‐voltage pulsed electricity from mechanical energy harvesters. However, the series connection of a large number of MSCs significantly reduces the overall capacitance and hence restricts their energy storage capability. It is therefore necessary for the individual MSCs to possess large areal capacitance to retain sufficiently high capacitance of the arrays without increasing the total footprint area. However, it is in general challenging to manufacture supercapacitors simultaneously possessing high‐rate capability and large areal capacitance.^[^ ^18^, ^19^, ^20^, ^21^ ^]^ Large areal capacitance often relies on thick electrodes, but thick electrodes typically feature lengthy and torturous ion transport pathways that degrade the electrochemical performance at high rates. The general way to eliminate this dilemma is to develop highly conductive thick electrodes with open,^[^ ^21^ ^]^ directional,^[^ ^19^ ^]^ and/or macroscopic^[^ ^20^ ^]^ pores, such as vertically aligned MXene flakes,^[^ ^19^ ^]^ macro‐porous MXene films^[^ ^20^ ^]^ and 3D carbon nanotube/graphene structures.^[^ ^18^, ^21^, ^22^ ^]^ The filling of electrolytes (in either liquid state or gel state) into these pore structures forms fast pathways for ion transport and high‐rate charge transfer at the electrode‐electrolyte interfaces to attain high‐rate performance. In particular, with directional pore structures penetrating from the top to the bottom of the electrodes,^[^ ^19^, ^21^ ^]^ high‐rate performance can be retained even when the electrode thickness exceeds 100 µm.^[^ ^19^ ^]^ However, in practice, such pore structures require complicated fabrication processes, thereby making it extremely challenging to manufacture these types of MSC arrays on a large scale.

Another way to prevent the series connection‐induced capacitance reduction is to widen the voltage window of individual MSCs so that much fewer MSCs are connected in series for the same overall voltage window of the array. In this setting, the emerging highly conductive pseudocapacitive electrode material MXene may not be favored due to its narrow voltage window of only 0.6 V in acid electrolytes. Although asymmetric MSCs may offer a wider voltage window of over 1.6 V by combining different types of electrode materials,^[^ ^23^ ^]^ they suffer from complicated fabrication processing and poor scalability. Ionic liquid‐based electrolytes can widen the voltage window to 3.0–4.0 V but are usually restricted to low‐rate capability^[^ ^24^ ^]^ <100 mV s^−1^.

To address these issues, we report a scalable full‐printing (for both electrodes and electrolytes) technique to manufacture metal‐free on‐paper MSCs simultaneously possessing a wide voltage window, large areal capacitance, and high‐rate capability. The key is the formulation of highly conductive organic inks based on properly doped poly(3,4‐ethylenedioxythiophene): poly(styrene sulfonate) (PEDOT:PSS) that enable scalable direct ink writing (DIW) of MSCs on paper substrates with excellent electrochemical performance. The printed symmetric MSCs simultaneously exhibit a wide voltage window of 1.6 V and a high areal capacitance over 60 mF cm^−2^ at a high scan rate of 1 V s^−1^. We have printed highly dense MSC arrays consisting of 100 cells connected in series on paper substrates, delivering a large overall capacitance of 3 µF at an ultrahigh scan rate of 30 V s^−1^ and an ultrawide voltage window of 160 V. The full‐printed MSC arrays can directly store the high‐voltage (>150 V) pulse electricity produced by droplet‐based electricity generators (DEGs)^[^ ^2^ ^]^ at a high energy storage efficiency of 62%. The totally eco‐friendly (metal‐free) on‐paper MSC arrays increase the potential for realizing sustainable self‐charging power systems for future electronic systems.

## 2 Results and Discussion

### 2.1 Scalable Fabrication of Thickness‐Independent High‐Rate MSCs

**Figure 1:**
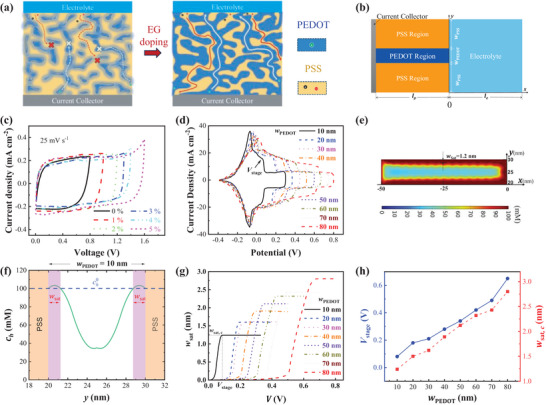
Effects of EG doping on the electrochemical performance of PEDOT: PSS‐based MSCs. a) Schematic of doping‐induced phase separation between PEDOT‐ and PSS‐rich regions. b) Schematic of the 2D two‐phase model for half‐cell MSCs. c) CV curves of the MSCs with PEDOT:PSS doped by EG of various *ϕ*_EG_. d) Simulated CV curves of the half‐cell MSCs with different *w*_PEDOT_. e) Simulated hole concentration distribution in the PEDOT region (*w*_PEDOT_ = 10 nm, *V* = 0.2 V). f) Simulated hole concentration profile at the position of *x* = –25 nm in (e). g) Dependence of *w*_sat_ on *V* for different *w*_PEDOT_. h) Dependence of potential window and critical saturation width *w*_sat,c_ on *w*_PEDOT_.

Based on PEDOT:PSS, we have developed a facile method to fabricate thickness‐independent high‐rate MSCs. In no need of any process to produce special pore structures, the simple DIW printing of properly doped PEDOT:PSS electrodes has enabled the MSCs to attain high‐rate capability >1 V s^−1^ for electrode thickness >100 µm. PEDOT:PSS is a conductive polymer incorporating hole‐conductive conjugated PEDOT of positive charges with electron‐insulating yet ion‐conductive PSS of negative charges. The insulating PSS increases the dispersibility of PEDOT:PSS in water^[^ ^25^ ^]^ and hence allows promising applications in photovoltaics,^[^ ^26^ ^]^ flexible electronics,^[^ ^27^, ^28^ ^]^ and energy storage devices.^[^ ^29^ ^]^ In addition, appropriate dopants, such as ethylene glycol (EG) and dimethyl sulfoxide (DMSO), can significantly increase conductivity of PEDOT: PSS by 2–3 orders of magnitude.^[^ ^25^ ^]^ Although the mechanism has not been fully understood yet, many studies support that the conductivity increase is due to the favorable doping‐induced phase separation to generate grown PEDOT‐rich grains which form interconnected highly hole‐conductive networks.^[^ ^26^, ^30^ ^]^ While at present the doping of PEDOT:PSS is mainly for conductivity increase,^[^ ^25^ ^]^ we have found that it also plays an important role in the electrochemical behavior of PEDOT:PSS‐based supercapacitors. In addition to the hole‐conductive PEDOT‐rich networks, the doping‐induced phase separation may simultaneously generate ion‐conductive PSS‐rich networks.^[^ ^30^ ^]^ The two types of networks may entangle with each other to form fast transport paths for both holes and ions to attain high‐rate capability even in very thick electrodes, as illustrated in **Figure** **1**a and to be demonstrated below. More importantly, our research has validated that sufficient doping can also significantly widen the voltage window of PEDOT:PSS‐based MSCs from the usual 0.8–1.0 V to 1.6 V.

To investigate the doping effects on the electrochemical performance of PEDOT:PSS electrodes, we use a mask‐based process to fabricate a set of MSCs, see Experimental Section, based on PEDOT: PSS dispersions in mixed water/EG solvents with the volume fraction of EG, *ϕ*_EG_, ranging from 0% to 30% (Figure 1c and Figure S1, Supporting Information). The MSC with pure (*ϕ*_EG_ = 0%) PEDOT:PSS electrodes exhibit a rectangular cyclic voltammetry (CV) curve over the voltage window of 0–0.8 V, consistent with the normal voltage window of PEDOT:PSS‐based supercapacitors.^[^ ^29^ ^]^ When the applied voltage is further increased beyond 0.8 V, the CV curve deforms evidently from the rectangular shape to endure a stage down at the high voltage region (Figure S1a, Supporting Information). When *ϕ*_EG_ increases from 0% to 5%, the voltage window (the voltage region of rectangular CV curves) gradually increases from 0.8 to 1.6 V. Further increasing *ϕ*_EG_ up to 30% does not increase the voltage window beyond 1.6 V, but it seems to be mainly limited by the electrolyte decomposition, rather than the stage down of the charging current (Figure S1b,c, Supporting Information). In order to understand the mechanism for doping‐widened voltage windows, we employ the 2D two‐phase model developed by Volkov et al.^[^ ^31^ ^]^ to conduct numerical simulations and investigate the dependence of the CV curves on the material morphology in the PEDOT:PSS electrodes. As illustrated in Figure 1b, the two‐phase model represents a nanoscale geometry of the PEDOT:PSS electrode which consists of the hole‐conductive PEDOT regions (of width *w*_PEDOT_) and the ion‐conductive PSS‐rich regions (of width *w*_PSS_). The model is detailed in Note S1, (Supporting Information). It has offered insight that the capacitance of PEDOT:PSS originates from the electrical double layers at the interface between the PEDOT‐ and PSS‐rich regions (hereafter denoted as P‐P interface) and their CV curves can be understood in terms of the coupled ion‐electron diffusion and migration.^[^ ^31^ ^]^ In this work, we have further found that the CV curves vary evidently with *w*_PEDOT_. As shown in Figure 1d, for different *w*_PEDOT_, all the CV curves in negative potential region remain unaltered, while in the positive potential region, they stage down at a certain potential, *V*_stage_. The wider PEDOT regions, the higher potential at stage down. Obviously, *V*_stage_ determines the voltage window of the MSCs. To unveil the mechanism of the stage down, we analyze the distribution of the hole concentration *c_h_* within the PEDOT region (Figure 1e–h; Figure S3, Supporting Information). During the charging process, *c_h_* increases with the applied potential *V*, thus generating the charging current, dominated by the temporal variation of *c_h_*. Consistent with previous research,^[^ ^25^, ^31^ ^]^ when *V* approaches *V*_stage_, *c_h_* at the P‐P interface becomes saturated (approaching the maximum concentration^[^ ^31^ ^]^ of accessible sites for holes $c_{h}^{0}$). However, the saturation region ($c_{h}=c_{h}^{0}\;$) has a finite width *w*_sat_ (Figure 1f). Further increasing *V* cannot increase *c_h_*, but increase *w*_sat_ (Figure 1g). Once *w*_sat_ reaches a critical width *w*_sat,c_, it also ceases to increase (Figure 1g), which significantly reduces the temporal variation of *c_h_* and causes the stage down of charging current. Because *w*_sat,c_ increase with *w*_PEDOT_ (Figure 1g), *V*_stage_ and hence voltage window should also increase with *w*_PEDOT_ (Figure 1d,h). Since the doping of PEDOT:PSS prompts the growth of PEDOT‐rich grains,^[^ ^25^, ^30^ ^]^ it can thereby widen the PEDOT‐rich regions and increase the voltage window of the MSCs. This explains our experimental results in Figure 1c.

It is of interest and importance to note that our simulations suggest that the voltage window of PEDOT:PSS electrodes is determined by the critical width *w*_sat,c_ of ≈1–3 nm (Figure 1h) at the P‐P interfaces. This further confirms the previous conclusion^[^ ^31^ ^]^ that the capacitance of PEDOT:PSS electrodes is dominated by the P‐P interfaces, rather than the electrode/electrolyte interface as in the general supercapacitors. Since the P‐P interfaces can be uniformly distributed inside the PEDOT:PSS electrodes (Figure 1b), their electrochemical performance is expected to be independent of the electrode thickness. In addition, besides widening the voltage window, the EG doping can greatly (by two orders of magnitude) increase the electronic conductivity of PEDOT:PSS only at the expense of slightly (by <2 times) reduced ionic mobility.^[^ ^30^ ^]^ One can then expect to use EG‐doped PEDOT:PSS to fabricate thickness‐independent high‐rate MSCs.

**Figure 2:**
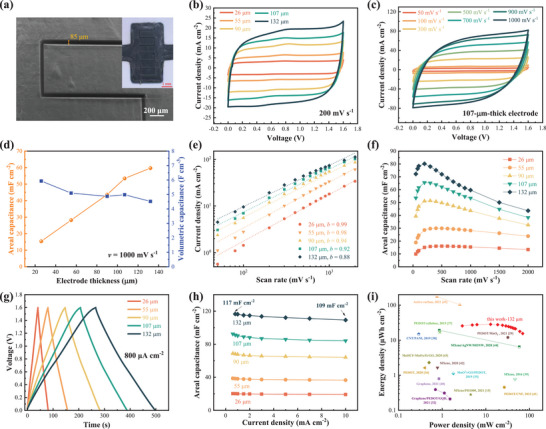
Morphology and Electrochemical characterization of the MSCs with DIW printed PEDOT:PSS electrodes. a) Top‐view SEM image of the MSC electrodes printed on a photo paper, (inset) photograph of the electrodes of an entire MSC (scale bar: 1 mm). b) CV curves of MSCs with various electrode thicknesses at the scan rate of 200 mV s^−1^. c) CV curves of an MSC with 107‐µm‐thick electrodes at different scan rates. d) Thickness‐dependent areal and volumetric capacitance at the scan rate of 1000 mV s^−1^. e) Log–log plot of the charging current density (at the voltage of 0.8 V) against scan rate. f) Areal capacitance extracted from the CV curves at various scan rates. g) GCD curves at the current density of 800 µA cm^−2^. h) Areal capacitance calculated from GCD curves at various current densities. i) Areal Ragone plot for the state‐of‐the‐art printed MSCs.

To fabricate thickness‐controllable MSCs and large‐scale MSC arrays, it is desired to develop a scalable printing technique for the PEDOT:PSS MSCs. However, the addition of EG into the PEDOT:PSS aqueous dispersion causes polymer aggregation that impairs printability. Following the strategy of our previous work,^[^ ^32^ ^]^ we also use electrochemically‐exfoliated graphene and graphene quantum dots (lateral size <20 nm) to stabilize the PEDOT:PSS water/EG dispersion without degrading the electrochemical performance. Initially, the EG concentration in the solvents is set as *ϕ*_EG_ = 20% to ensure a wide voltage window and high‐rate capability of the MSCs and meanwhile retain suitable properties (especially viscosity and drying speed) of the final inks for the printing process. We further use vacuum drying to concentrate and densify the PEDOT:PSS inks for effective DIW of thick MSC electrodes. The inks are highly stable and there is no occurrence of any polymer aggregation even when the weight concentration of all the solid materials increases up to 3%. The ink viscosity increases with the concentration and exceeds 1000 Pa s at the concentration of 3% (Figure S4, Supporting Information). The higher viscosity gives rise to higher resolution of the printed patterns. The 3% ink enables direct printing of fine patterns with a lateral resolution ≈100 µm and a thickness ≈5 µm for one printing pass (with 25 printing passes, the pattern thickness is over 130 µm, Figure S5, Supporting Information). The electrical conductivity is nearly independent of the thickness, varying within a narrow range between 225 to 322 S cm^−1^ (Figure S6, Supporting Information). However, for thick patterns, the lateral resolution decreases due to the need for multiple printing passes. To overcome the shortcoming, we combine the DIW with a femtosecond laser scribing process to fabricate thick MSC electrodes on paper substrates with high lateral resolution in a time‐ and material‐effective manner. As illustrated in Figure S7 (Supporting Information), after printing the non‐gapped MSC electrodes, a femtosecond laser is used to scribe the electrodes into interdigitated structure (**Figures** **2**a and S8, Supporting Information). Thanks to the high‐resolution femtosecond laser scribing process,^[^ ^33^ ^]^ the inter‐finger gap in our MSCs is as narrow as 85 µm even when the electrode thickness is 105 µm, corresponding to a high aspect ratio of >1.2. The narrow gaps shorten the transport distance of the electrolyte ions and further contribute to the high‐rate capability of the MSCs.^[^ ^34^ ^]^ Meanwhile, they minimize the waste of active materials and energy because only a small amount of the electrode materials within the gap regions are removed by laser.

The scalable fabrication of small‐size (footprint area down to 2–3 mm^2^) and thick (up to >100 µm) MSCs enables us to investigate their thickness‐dependent electrochemical performance. At a medium scan rate of 200 mV s^−1^ (Figure 2b), the MSCs exhibit ideal capacitive behavior as indicated by the nearly perfect rectangular shape of the CV curves over the wide voltage window of 1.6 V for all the electrode thickness, ranging from 26 µm for 5 printing layers to 132 µm for 25 printing layers. Even at the high scan rate of 1 V s^−1^, the CV curves of all the MSCs still exhibit excellent rectangularity (Figures 2c and Figure S9c, Supporting Information), indicating excellent rate capability^[^ ^19^ ^]^ in spite of the use of gel electrolytes, mixture of poly(4‐styrenesulfonic acid) (PSSH) and H_3_PO_4_, that have relatively lower ionic conductivity than the liquid‐state electrolytes often used in other high‐rate supercapacitors.^[^ ^18^, ^19^, ^20^, ^22^ ^]^ As the thickness increases, the areal capacitance increases linearly, while the volumetric capacitance merely declines slightly (Figure 2d). Figure 2e plots the charging current density *i* (at the voltage of 0.8 V) against the scan rate *v*, following the power law *i* ∼ *v^b^* with *b* being the characteristic exponent.^[^ ^19^ ^]^ At the scan rates between 200 and 2000 mV s^−1^, *b* is very close to 1 for the MSCs with electrodes no thicker than 55 µm and still ≈0.9 for the thickest (132 µm) MSC. These suggest that the charge‐storage kinetics of all our MSCs are dominated by the high‐rate surface/interface interactions, not the slow diffusion‐limited bulk interactions,^[^ ^19^ ^]^ and the high‐rate performance is almost independent of electrode thickness. Moreover, one should note that our MSC electrodes are based on very dense (non‐porous) PEDOT:PSS films (Figure 2a and Figure S5, Supporting Information). Morphologically, there is no significant amount of electrode/electrolyte interfaces to facilitate high‐rate charge storage. Once more, it can be inferred that the doping‐induced phase separation in PEDOT:PSS films generates an enormous number of internal P‐P interfaces, which leads to the thickness‐independent high‐rate capability of the MSCs. As confirmed in Figure 2f, with the scan rate increasing from 200 to 1000 mV s^−1^, the areal capacitance remains constant for the 26 and 55 µm‐thick MSCs and only declines slightly (≈15%) for the 107‐µm‐thick MSC. Similar to our previous work,^[^ ^32^ ^]^ the MSCs exhibit a low‐rate decay: when the scan rate <200 mV s^−1^, the capacitance decreases with decreasing scan rate (Figure 2f). The mechanism has not been clear but is likely related to the interaction between PEDOT:PSS, GQDs, and EG doping according to our previous work^[^ ^32^ ^]^ and Figure S1e (Supporting Information) in this work. Nevertheless, the galvanostatic charge‐discharge (GCD) curves of all the MSCs are symmetrical and triangular at a high current density from 0.8 mA cm^−2^ (Figure 2g) to 10 mA cm^−2^ (Figure S9a, Supporting Information), demonstrating a high coulombic efficiency. The highest areal capacitance obtained from the GCD curves (Figure 2h) reaches 117 mF cm^−2^ for the 132 µm MSC at 0.8 mA cm^−2^, and remains 109 mF cm^−2^ even at 10 mA cm^−2^, confirming the excellent rate capability at large electrode thickness. Compared with most of the printed MSCs (Table S2, Supporting Information), our MSCs possess a wider voltage window, larger areal capacitance, higher rate capability, and hence much higher energy density and power density. Specifically, the areal energy density of the 132‐µm‐thick MSC reaches 28.5 µWh cm^−2^ at the power density of 12.8 mW cm^−2^ (at the scan rate of 200 mV s^−1^), or 15.5 µWh cm^−2^ at 70 mW cm^−2^ (at the scan rate of 2000 mV s^−1^), significantly surpassing most of the state‐of‐the‐art printed MSCs (Figure 2i and Table S2, Supporting Information).^[^ ^15^, ^29^, ^32^, ^35^, ^36^, ^37^, ^38^, ^39^, ^40^, ^41^, ^42^, ^43^, ^44^, ^45^ ^]^

### 2.2 Large‐scale MSC arrays

**Figure 3:**
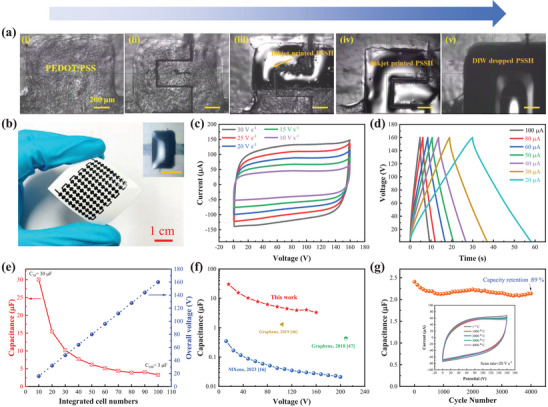
Fabrication and characterization of a large‐scale (100‐cell) MSC array. a) Optical micrographs indicating the scalable fabrication process of individual MSCs: i) DIW printing of the non‐gapped MSC electrode, ii) laser scribing into interdigitated electrodes, iii) during and iv) after the inkjet printing of low‐concentration PSSH electrolyte, and v) after DIW deposition of a drop of high‐concentration PSSH electrolyte. b) Photographs of the 100‐cell MSC array on photopaper, (inset) photograph of one individual MSC cell (scale bar: 1 mm). c) CV curves at the scan rate from 10 to 30 V s^−1^. d) GCD curves at the current from 20 to 100 µA. e) Cell number‐dependent overall capacitance and voltage window. f), Capacitance against voltage window for various MSC arrays. g) Cycling stability for 4000 CV cycles at 20 V s^−1^, (inset) CV curves after different number of cycles.

The stable high‐concentration PEDOT:PSS inks and the scalable printing process enable us to monolithically fabricate a large‐scale MSC array comprising up to 100 series‐connected cells within a small footprint area (2.4 × 3.4 cm^2^, **Figure** **3**b). As illustrated in Figure S7 (Supporting Information), first, the array pattern, including the 100 individual MSC cells (each of area as small as 1.2 × 2.2 mm^2^, Figure 3a(i), and the interconnect, was printed on paper with the PEDOT:PSS inks through DIW. Then, after all the MSC electrodes were scribed via the femtosecond laser into interdigitated structures (Figure 3a(ii) and Figure S10a, Supporting Information), the PSSH/H_3_PO_4_ ink was printed on every MSC cell to form the gel electrolyte. However, the gel electrolytes need to be printed in large thickness (to ensure the high‐rate performance of the MSCs) and exactly within the MSC electrode area (to avoid electrolyte short with the neighboring MSCs where the inter‐cell distance is only as small as 1 mm). It is challenging for DIW to “efficiently” print such small thick electrolyte patterns^[^ ^16^ ^]^ due to the inevitable tradeoff between resolution and time efficiency. To address the challenge, we combine DIW with inkjet printing to develop a self‐assembly process to realize high‐resolution and high‐efficiency fabrication of the electrolyte patterns. Considering the surfaces of both the commercial paper substrates and the PEDOT:PSS electrodes are hydrophobic, we first use inkjet printing to precisely print a thin layer of PSSH/H_3_PO_4_ electrolyte on the MSC electrodes to modify their surface to become hydrophilic (Figure 3a(iii,iv)). Then, on the center of every MSC, a drop of high‐concentration PSSH/H_3_PO_4_ electrolyte ink is deposited through DIW and spreads to form self‐assembled thick small electrolytes (Figure 3a(v),b (inset) and Figure S10b, Supporting Information).

The CV curves of the 100‐cell MSC arrays exhibit perfect rectangularity at the ultrahigh scan rate of up to 30 V s^−1^ (Figure 3c) within a remarkably wide voltage window of 160 V. The capacitance retains ≈90% after 4000 CV cycles at 20 V s^−1^ (Figure 3g), confirming the high‐rate capability and good cycle life. It is worth mentioning that here the highest scan rate is likely restricted by our characterization equipment, rather than the upper limit of rate capability of the MSC arrays themselves. Their GCD curves also indicate ideal symmetric triangles with negligible IR drop undercurrents of up to 100 µA (Figure 3d). A fine study (Figure 3e and Figure S11, Supporting Information) confirms that with increasing the cell number, the voltage window increases in proportion, and the overall capacitance decreases in reverse proportion, confirming the excellent scalability of our fabrication process. The capacitance (3 µF) of the 100‐cell array is almost one order of magnitude higher than that of the previously reported MSC arrays with voltage window beyond 100 V (Figure 3f and Table S3, Supporting Information).^[^ ^16^, ^46^, ^47^ ^]^ The improved capacitance of our large‐scale MSC arrays can be ascribed to the simultaneously attained large areal capacitance and wide voltage window of the individual MSCs. The former ensures large capacitance of the arrays, while the latter reduces the number of needed MSCs in the series connection (Figure S12, Supporting Information) to further minimize the capacitance reduction. Our metal‐free on‐paper MSCs array also demonstrates outstanding flexibility, with nearly unaltered CV curves under various bending angles (Figure S13, Supporting Information).

### 2.3 Direct Storage of High‐Voltage Pulse Electricity from DEGs

**Figure 4:**
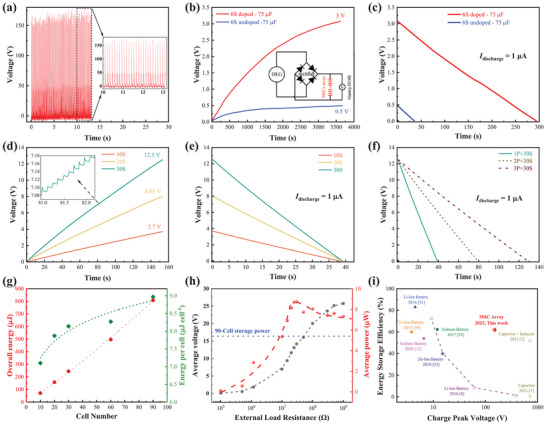
Energy storage efficiency of the MSC arrays charged by the DEG. a) Output voltage signals of the DEG after rectification by a bridge rectifier, (inset) close‐up view of the output voltage. b) Charging (by the DEG) curves of two 6‐cell arrays of doped and undoped PEDOT:PSS MSCs, (inset) circuit diagram of the charging test. c) Galvanostatic discharging curves of the two arrays charged by DEG in (b). d) Charging (by the DEG) curves of the MSC arrays with different cell numbers. e) Galvanostatic discharging curves of the three arrays charged by DEG in (d). f) Galvanostatic discharging curves of three MSC arrays with combined parallel‐series modes of 1P × 30S, 2P × 30S, and 3P × 30S, which have been charged by the DEG for 150 s in the pure series connection mode of 3S, 60S and 90S, respectively. g) Total energy stored in the arrays and average energy stored in individual cells as a function of the cell number after the MSC arrays are charged by the DEG for 150 s. h) The measured average voltage and power of the DEG under different external load resistances. Blue dash line indicates the storage power of the 90‐cell MSC array. i) Comparisons of peak voltage‐dependent energy storage efficiency between various energy storage devices. All the devices were charged by pulse electricity produced by mechanical energy harvesters.

We evaluate the efficiency of our MSC arrays for storing electricity produced by a DEG (Figure S14, Supporting Information).^[^ ^2^ ^]^ All the test conditions for the DEG are kept constant. The DEG produces pulse electricity with an open‐circuit peak voltage of 150 V (**Figure** **4**a) and a short‐circuit current ≈200 µA (Figure S14b, Supporting Information). To effectively capture such instantaneous electricity, ultrahigh‐rate performance of the energy storage devices is highly desired. To verify this point, we have fabricated two types of 6‐cell MSC arrays on paper substrates, one based on the high‐rate doped PEDOT:PSS electrodes and the other on the pristine (undoped) PEDOT:PSS electrodes (Figure S15, Supporting Information). Although both MSCs arrays exhibit rectangular CV curves and comparable capacitance (≈75 µF) at the low scan rate of 0.3 V s^−1^ when the scan rate increases to 6 V s^−1^, only 7% capacitance is retained in the undoped PEDOT: PSS MSC array while 88% retains in the doped one (Figure S15b,c, Supporting Information). After being charged by the DEG for 1 h, the doped MSC array reached a voltage of 3.0 V while the undoped array could only attain 0.48 V (Figure 4b). Afterward, the two MSC arrays were discharged at the same current of 1 µA. It took 294 s for the doped array to fully discharge (from 3.0 to 0 V), while only 36 s for the undoped array (Figure 4c). Correspondingly, with the same charging time, the doped array stored an energy of 455 µJ, ≈50 times more than the undoped array (8.6 µJ), confirming that the high‐rate capability of the doped PEDOT:PSS MSCs significantly improves the storage efficiency of the instantaneous electricity.

The energy storage efficiency can be further improved by increasing the cell number of the MSC arrays. After being charged by the DEG for the same period of 150 s, the attained voltage of the MSC arrays (Figure S16, Supporting Information) increases with their cell number, from 3.7 V for 10 cells to 12.5 V for 30 cells. The stepwise voltage increase (inset of Figure 4d) is similar to the energy storage behavior charged by a piezoelectric energy harvester,^[^ ^48^ ^]^ confirming the charging of MSC arrays by instantaneous (pulsed) electricity. When discharging at the same current of 1 µA, the voltage of all the MSC arrays decreases linearly with time (Figure 4e). Following the principle of “charging in series and discharging in parallel” that is often implemented for the energy storage units in self‐charging power systems,^[^ ^49^ ^]^ we use the DEG to charge the MSC arrays of 30, 60 and 90 cells in series connection also for 150 s, but discharge them in the combined parallel‐series connection mode of 1P × 30S, 2P × 30S, and 3P × 30S, respectively (here “*m*P× *n*S” means parallel connections of *m* groups of *n* series‐connected cells). Before discharge, all the combined MSC arrays attain approximately the same initial voltage (Figure 4f). At the same constant current of 1 µA, the discharging time of the MSC arrays is nearly proportional to the cell number, suggesting the excellent scalability of our MSC arrays. The discharge behaviors are used to evaluate the energy stored in the MSC arrays (Figure 4g and Table S4, Supporting Information). It is clear that after being charged by the DEG for the same time of 150 s, the total energy stored at the MSC arrays increases drastically with the cell number. Even the average energy stored in one individual cell also increases evidently with the total cell number (Figure 4g). The phenomena should be mainly ascribed to the significantly increased voltage window and charging rate (both proportional to the cell number) in the MSC arrays so that an MSC cell in a large‐scale array can store more energy from the pulse electricity than in a small‐scale array. To confirm the high energy storage efficiency (ESE) of the MSC arrays, we measured the average voltage^[^ ^50^ ^]^ and the average output power of the DEG under various external load resistances ranging from 10 kΩ to 1000 MΩ (Figure 4h). The average power reaches the maximum value of 8.7 µW at 30 MΩ. The 90‐cell array stores in total 807.6 µJ within 150 s, corresponding to an effective storage power of 5.4 µW, 62% of the maximum average output power of the DEG. In other words, when storing pulse electricity with a high peak voltage of 150 V, the ESE of our 90‐cell MSC array is as high as 62%. As shown in Figure 4i, the previous studies^[^ ^7^, ^8^, ^12^, ^51^, ^52^, ^53^, ^54^, ^55^ ^]^ have demonstrated that when storing pulsed electricity, the ESE of the present devices (batteries or capacitors) decays drastically with the peak charging voltage. When the charging peak voltage is >100 V, the ESE is only 0.04% for capacitors (at ≈750 V)^[^ ^7^ ^]^ and 1.2% for Li‐ion batteries (at ≈400 V).^[^ ^8^ ^]^ Certainly, it is possible to increase the ESE by employing additional components (such as inductors,^[^ ^7^ ^]^ capacitors,^[^ ^11^ ^]^ and transformers^[^ ^8^, ^12^ ^]^) in the power management circuits, but this inevitably increases the complexity and form factor of the systems. In this sense, the 90‐cell MSC arrays are superior thanks to their simplicity (in no use of any auxiliary components) and compactness (footprint area as small as 2.4 × 3.4 cm^2^ and total thickness ≈300 µm, including the thickness of the paper substrate and electrolyte).

Finally, as a demo, we use the DEG‐charged MSC arrays to light up LEDs. The LED powered by the 30‐cell MSC array is much brighter than that directly powered by the DEG (Figure S17, Supporting Information). After being charged by the DEG for the same time of 150 s, the 10‐cell MSC array can continuously light up the LED for 1.8 s while the 30‐cell array for 6.0 s (Video S1, Supporting Information), further confirming the increase of ESE with the cell number.

## 3 Conclusion

In conclusion, based on doped PEDOT:PSS, we have developed a simple and scalable process to monolithically print ultrahigh‐rate large‐scale metal‐free MSC arrays on paper substrates to directly store the high‐voltage (>100 V) instantaneous (pulse) electricity produced by recent mechanical energy harvesters. The individual MSCs exhibit nearly thickness‐independent electrochemical performance and simultaneously possess a wide working voltage window of 1.6 V, a large areal capacitance up to >60 mF cm^−2^, and a high rate capability of 1 V s^−1^. Computer simulations based on the 2D two‐phase model developed by Volkov et al. have suggested that the wider working voltage window should be ascribed to the doping‐induced widening of the PEDOT‐rich regions (grains) that possess wider hole saturation region at the interfaces between PEDOT‐ and PSS‐rich regions. The large‐scale MSC arrays consisting of up to 100 cells can be fully printed on paper substrates within a small footprint area of 2.4 × 3.4 cm^2^. Thanks to their striking performance of large capacitance >3 µF, ultrawide working voltage window up to 160 V, and ultrahigh rate capability over 30 V s^−1^, the MSC arrays can directly store instantaneous high‐voltage (>150 V) electricity with a high energy storage efficiency of 62%, over one order of magnitude higher than that of the present batteries and capacitors. The on‐paper, metal‐free, and large‐scale MSC arrays provide an efficient, compact, and sustainable solution to address the challenge of directly storing the high‐voltage instantaneous electricity produced by mechanical energy harvesters and will contribute to the development of entirely sustainable self‐charging power systems for emerging electronics.

## 4 Experimental Section

### Formulation of PEDOT: PSS Ink for DIW

The active materials in DIW inks comprise PEDOT:PSS (1.1 wt% water dispersion, Product No. 739 332, Sigma‐Aldrich), electrochemically exfoliated graphene (EEG), and graphene quantum dots (GQD). The EEG and GQD were synthesized according to the previous publications.^[^ ^13^, ^32^ ^]^ First, 10 mL EEG/Dimethylformamide (DMF) dispersion (15 mg mL^−1^) was centrifuged at 3000 rpm for 5 min to remove the large graphene particles, and the harvested supernatant was further centrifuged at 13 000 rpm for 30 min to separate the fine and few‐layer EEG from the DMF. Second, the harvested EEG (≈40 mg) and 10 mg GQD were added into the mixture of 8 mL PEDOT: PSS and 2 mL ethylene glycol (99.5%, Product No. 1.09621, Sigma‐Aldrich). The dispersion was sonicated for 30 min and stirred overnight to form a uniform ink. Finally, the ink was dried under vacuum at room temperature to attain the viscous inks with a solid content of 3 wt.% for DIW.

### MSC Fabrication

All the MSCs were fabricated on paper substrates, either on photopaper (HP, CR757A, glossy photo paper) or carton paper (Pure‐Pak classic carton for milk boxes).

### Mask‐based MSC Fabrication

To investigate the effect of EG doping on the voltage window of the PEDOT:PSS‐based MSCs, a mask‐based process was used to fabricate the MSCs. First, for every doping concentration, a certain volume of EG was added into 0.5 mL PEDOT:PSS aqueous dispersion to attain the proper EG volume fraction *ϕ*_EG_ (ranging from 0% to 30%), followed by magnetic stirring for 10 min. Second, a Kapton tape was patterned into a mask with an opening (consisting of one center rectangle of 3 × 6 mm^2^ and two extended rectangles of 6 × 1.5 mm^2^ as the leads for external electrical connection, as shown in Figure S1d, Supporting Information) by CO_2_ laser (Universal Laser Inc.). The Kapton mask was pasted on a photopaper substrate, and 30 µL EG‐doped PEDOT:PSS dispersion was dropped into the opening. After the dispersion dried, the Kapton mask was removed, and the remaining PEDOT:PSS at the center rectangle region was scratched by a knife into a pair of symmetric electrodes for the MSC. Finally, 10 µL high concentration electrolyte (a mixture of PSSH and H_3_PO_4_ with a volume ratio of 3.6: 1) was dropped on top of the center electrodes and dried to form a symmetric MSC for CV tests.

### DIW Printing of MSCs

The DIW printing procedure included 3 steps, printing of non‐gapped electrodes, laser scribing into the interdigitated electrodes, and electrolyte deposition. For the non‐gapped electrode printing, the 3 wt.% PEDOT:PSS inks were printed with the FELIX BIO printer (FELIXprinters, Netherlands) equipped with a 10 mL syringe and different diameter nozzles (200 µm for printing of MSC electrodes, 100 µm for printing of interconnects) on the photopaper or carton paper at the substrate temperature of 80 °C.

Then, a sub‐picosecond laser (Spirit 1040‐4‐SHG, Spectra‐Physics of Newport Corporation) with a central wavelength of 520 nm was used to scribe the DIW‐printed non‐gapped electrodes into interdigitated electrodes. A suitable laser power setting of 600 mW with repetition rate of 10 kHz was applied to ensure sufficient scribing of the electrode materials without damaging the paper substrate. During the scribing, a 3‐axis linear motorized stage (XMS100, Newport) was used to move the MSCs at the speed of 500 µm s^−1^.

Finally, the PSSH electrolytes were printed. There were two types of PSSH electrolyte inks for DIW and inkjet printing, respectively. The former was of high concentration and prepared through mixing 0.5 mL poly(4‐styrenesulfonic acid) solution (PSSH, *M*_w_ ≈ 75 000, 18 wt.% in H_2_O, product number: 561223, Sigma‐Aldrich) with 0.14 mL phosphoric acid (H_3_PO_4_, ≥85%, Sigma‐Aldrich, product number: 40 278). The latter was of low concentration and prepared by mixing 0.2 mL PSSH with 0.5 mL deionized water, 0.5 mL ethylene glycol, and 0.14 mL H_3_PO_4_. For individual MSCs, the high‐concentration PSSH electrolyte ink was drop cast onto the electrodes and dried to form the gel electrolytes. For the MSC arrays, first, a commercial piezoelectric Dimatix Materials printer (DMP 2800, Dimatix‐Fujifilm Inc.), equipped with a 10 pL cartridge (DMC‐11610) was employed to print 4 layers of the low‐concentration PSSH electrolyte ink to cover the interdigitated electrodes at a drop spacing of 30 µm. Second, for every MSC cell, 2 µL high‐concentration PSSH electrolyte ink was deposited into the center of the inkjet‐printed PSSH area with the FELIX BIOprinter and spread evenly over the entire inkjet‐printed area. Finally, the printed PSSH electrolytes dried at fume hood overnight to form well‐patterned (self‐assembled) gel electrolytes for the MSC arrays.

### DEG Fabrication and Characterization

The DEG was fabricated according to the previous research.^[^ ^56^ ^]^ A 3  × 4 cm^2^ Polytetrafluoroethylene film (PTFE, Sigma‐Aldrich, 100 µm thick) was cleaned with acetone and deionized water. Then a piece of conductive copper tape with the same size was pasted on its back side as the bottom electrode. A thin aluminum tape was pasted on its front side as the top electrode. Both electrodes individually connected to an external circuit by copper wires for measurement. A disposable infusion set (Jiangxi Fenglin Medical Appliances Co., Ltd, China) was used as the droplet generator and the working condition was kept constant throughout the research (droplet volume 50 µL, impinging height 10 cm, PTFE sliding angle 35°, and impinging frequency 9 Hz).

The output voltage of DEG was measured by the oscilloscope (RSDS 1152CML+, RSPRO, Sweden) with a high‐impedance (100 MΩ) probe after a rectifier (200 V, 0.8 A, MiniDIP SMD4, China). The output current of the DEG was measured using a modified shunt‐resistor method, in which DEG connects in series with a 10 kΩ load resistor and 100 Ω shunt resistor. The oscilloscope was used to measure the voltage drop across the shunt resistor, and the current of DEG could be calculated from the obtained voltage by using Ohm's law.

### Electrochemical Measurement and Calculation

All the electrochemical measurements of the individual MSCs and MSC arrays, including cyclic volumetry (CV), galvanostatic charge–discharge (GCD) and electrochemical impedance spectroscopy (EIS) were carried out in a two‐electrode system using an electrochemical working station Gamry Interface 1010E (Gamry Instruments Inc., Warminster PA, USA) connected with a Signatone S‐1160 probe station equipped with S‐725 micropositioners (Signatone Corporation, Gilroy CA, USA) when the voltage window was no >12.5 V. For voltage window >12.5 V, the CV and GCD measurements were conducted via a Keithley 4200A‐SCS parameter analyzer (Tektronix, Inc.) connected to a shielded wafer prober (Cascade Microtech Summit 11 000). Before the test, silver paste was applied to the two leads of MSCs and arrays for the external connection. All the areal capacitance was calculated based on the CV or GCD curves. For the CV curves, the following equation is used,

(1) $$C_{A,\mathrm{CV}}=\frac{\int \left(i_{C}-i_{d}\right)dv}{2vAV}$$

where *V*, *v*, *i_c_*, *i_d_* and *A* refer to the voltage window, scan rate, charging current, discharging current, and the footprint area of the devices (including the inter‐finger gaps), respectively.

For the GCD curves, the following equation is used,

(2) $$C_{A,\mathrm{GCD}}=\frac{I\Delta t}{AV}$$

where *I* and Δ*t* refer to the discharging current and discharging time, respectively.

The areal energy density (*E_A_*) and power density (*P_A_*) of the MSCs were calculated as the following equations,

(3) $$E_{A}=\frac{C_{A,\mathrm{CV}}V^{2}}{2}$$

(4) $$P_{A}=\frac{E_{A}}{V/v}$$

When the MSC arrays were charged by the DEG, the overall stored energy (*E_h_*) was calculated by the discharge curves with

(5) $$E_{h}=\frac{1}{2}It_{d}V_{d}$$

where *I*, *t*_d_ and *V*_d_ refer to the discharging current, discharging time, and discharging voltage window, respectively.

To quantify the ability to store/capture the high‐power instantaneous electricity of our MSC arrays, the average voltage *U*_RMS_ and average output power *P*_DEG_ of DEG under different external load resistances were calculated. The test circuit diagram is in Figure S18 (Supporting Information).

(6) $$U_{\mathrm{RMS}}=\sqrt{\frac{\int U{\left(t\right)}^{2}dt}{T}}$$

(7) $$U_{\mathrm{PEG}}=\frac{{\left(U_{\mathrm{RMS}}\right)}^{2}}{R}$$

where *U*(*t*), *T* and *R* refer to the measured voltage over time, the total time of the measurement, and the total resistance, respectively. The total resistance was calculated by connecting the external load resistance in parallel with the internal resistance (100 MΩ) of the oscilloscope.

### Material Characterizations

The surface morphology of the printed PEDOT:PSS electrodes were characterized by SEM (Gemini Ultra 55, Zeiss, Germany). The viscosity of the DIW inks was measured by using a rotational rheometer (DHR‐2, TA Instrument) with 25‐mm diameter steel parallel‐plate geometry at 25 °C.

## Conflict of Interest

The authors declare no conflict of interest.

## Supporting information

Supporting Information

Supplemental Video 1

## Data Availability

The data that support the findings of this study are available from the corresponding author upon reasonable request.
