# Supplementary material for: Ultrafast Metal‐Free Microsupercapacitor Arrays Directly Store Instantaneous High‐Voltage Electricity from Mechanical Energy Harvesters
Source: Adv Sci (Weinh). 2024 Mar 19;11(22):2400697. doi: 10.1002/advs.202400697 (PMC11165484; doi:10.1002/advs.202400697)
Supplement: Supplementary file 1 — Supporting Information [file ADVS-11-2400697-s001.pdf]

ADVANCED SCIENCE
Open Access

Supporting Information

for *Adv. Sci.*, DOI 10.1002/advs.202400697

Ultrafast Metal-Free Microsupercapacitor Arrays Directly Store Instantaneous High-Voltage Electricity from Mechanical Energy Harvesters

*Shiqian Chen, Zheng Li, Po-Han Huang, Virginia Ruiz, Yingchun Su, Yujie Fu, Yolanda Alesanco, B. Gunnar Malm, Frank Niklaus and Jiantong Li**

Supporting Information

Ultrafast Metal-Free Microsupercapacitor Arrays Directly Store Instantaneous High-Voltage Electricity from Mechanical Energy Harvesters

Shiqian Chen[†], *Zheng Li*[†], *Po-Han Huang, Virginia Ruiz, Yingchun Su, Yujie Fu, Yolanda Alesanco, B. Gunnar Malm, Frank Niklaus, Jiantong Li*[*]

S. Chen, Z. Li, Dr. Y. Su, Y. Fu, Prof. B. G. Malm, Prof. J. Li

KTH Royal Institute of Technology, School of Electrical Engineering and Computer Science, Division of Electronics and Embedded Systems, Electrum 229, 16440 Kista, Sweden.

[†] These authors contributed equally to this work.

[*] Corresponding author: Jiantong Li, jiantong@kth.se

Dr. P.-H. Huang, Prof. F. Niklaus

KTH Royal Institute of Technology, School of Electrical Engineering and Computer Science, Division of Micro and Nanosystems, Sweden.

Dr. V. Ruiz[‡], Dr. Y. Alesanco

CIDETEC, Basque Research and Technology Alliance (BRTA), Po. Miramón 196, 20014 Donostia-San Sebastián, Spain.

[‡] Present address: International Research Center in Critical Raw Materials-ICCRAM, Universidad de Burgos, Plaza Misael Bañuelos s/n, E-09001, Burgos, Spain.

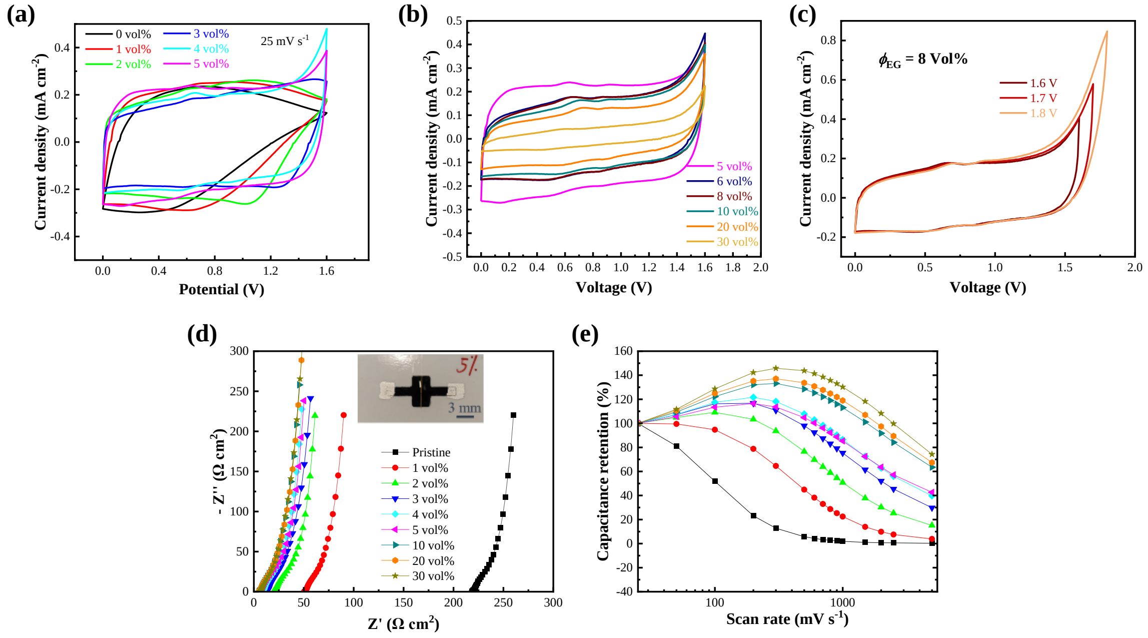

Figure S1: Electrochemical performance of MSCs fabricated through mask-based process with PEDOT:PSS dispersions doped with EG at different ϕ_{EG}. a-c, CV curves (at the scan rate of 25 mV s^{-1}). **d**, Nyquist plot, (inset) Photograph of the MSC with ϕ_{EG} = 5 vol%. **e**, Capacitance retention versus scan rates.

Supplementary Note 1.

2D two-phase model for PEDOT: PSS

To gain more insight into dependence of the CV curves and working voltage window (WVW) of the MSCs on the material morphology in the PEDOT:PSS electrodes (especially the width of the PEDOT-rich region), we employ the 2D two-phase model[1] developed by Volkov *et al*. to conduct computer simulations. As illustrated in Fig. 1b, the two-phase model represents a nano geometry of the PEDOT:PSS electrode (of thickness l_p) which consists of the electronically-conductive PEDOT regions (of width w_{PEDOT}) and the ionically-conductive PSS-rich regions (of width w_{PSS}). The metal current collector and electrolyte are located on the left and right sides, respectively. The electron (hole) and ion transport inside the two-phase electrode is modeled with the modified Nernst-Plank-Poisson approach[1]. The boundary conditions are constrained based on the assumption that only holes transport in the PEDOT region, while only ions transport in the PSS and the electrolyte regions, as detailed below.

There are three types of chemical species in the systems, hole (h), cations (+) and anions (–). Their flux densities are described by the continuity equation (S1)

$$\frac{\partial c_i}{\partial t} + \vec{\nabla} \cdot \vec{j_i} = 0 \qquad (\mathbf{S1})$$

and the Poisson's equation (S2)

$$-\epsilon_0 \epsilon_r \Delta V = F(c_+ - c_- + c_h - c_{\text{fixed}}) \qquad (\mathbf{S2})$$

where c_i and $\vec{j_i}$ are respectively the concentration and flux of a chemical specie with *i* being *h*, + or –, *t* is time, F is the Faraday constant, V is the electric potential, ϵ_0 is the permittivity of the vacuum, $\epsilon_r = 81$ is the dielectric permittivity of the system, and c_{fixed} = 1 mM is the concentration of residual negative ions on the PSS chains and only exists in the PSS regions.

In the PEDOT-rich region, only holes can transport, and the hole flux $\overrightarrow{J_h}$ obeys the modified Nernst-Planck equation as

$$\overrightarrow{J_h} = -D_h \vec{\nabla} c_h - f D_h c_h \vec{\nabla} V \left(1 - \frac{c_h}{c_h^0}\right) \qquad (\mathbf{S3})$$

where c_h is hole concentration, the additional factor $(1 - \frac{c_h}{c_h^0})$ is to ensure $c_h \leq c_h^0$ with c_h^0 being the concentration of accessible sites of holes, D_h is the hole diffusivity in PEDOT, $f = \frac{F}{RT}$ (*R* is the molar constant, and *T* = 300 K is the temperature). The boundary conditions for Eq. (S1) and (S3) in the PEDOT region are specified in Figure S2a.

In the PSS-rich region and electrolyte region, only ions can transport so that modified Nernst-Planck equation reads as

$$\overrightarrow{J_\pm} = -D_\pm \vec{\nabla} c_\pm - f D_\pm c_\pm \vec{\nabla} V - D_\pm \frac{c_\pm \vec{\nabla}(c_+ + c_-)}{c_{\text{max}} - (c_+ + c_-)} \qquad (\mathbf{S4})$$

where $D_\pm$ is the diffusivity of the cations (+) and anions (–), c_{max} is the maximum ion concentration in the regions. The last term in Eq. (S4) is a correction because of the finite size of ions[1]. The boundary conditions for Eq. (S1) and (S4) in the PSS and electrolyte regions are specified in Figure S2b. Note that the continuity boundary condition for ion concentrations holds at the interface between PSS regions and electrolyte.

Finally, throughout all the regions, the hole/ion concentrations are correlated to the potential distribution according to the Poisson's equation (S2). The boundary conditions for Eq. (S2) specified in Figure S2c. Note that the continuity boundary condition for potential *V* holds at all the internal interfaces (PEDOT-PSS, PEDOT-electrolyte, and PSS-electrolyte). The potential applied at the metal current collector is

$$V = V_{\text{in}}(t) = \begin{cases} V_{\min} + vt & (t \le t_0) \\ V_{\max} - v(t - t_0) & (t > t_0) \end{cases},$$

where $V_{\min}$ and $V_{\max}$ are the minimum and maximum applied potential, respectively, v is the scan rate, and $t_0 = (V_{\max} - V_{\min})/v$ is the half cycle period. With the applied $V_{\text{in}}(t)$, Eq. (1)-(4) can be self-consistently solved to get the distribution of c_h, $c_{\pm}$, and V. The current density is obtained by integration over the PEDOT region as

$$j(t) = \frac{F}{2w_{\text{PSS}} + w_{\text{PEDOT}}} \iint \frac{\partial c_h}{\partial t}\, ds. \tag{S5}$$

The plot of $j(t)$ against $V_{\text{in}}(t)$ gives the simulated CV curves.

The simulation was carried out through COMSOL Multiphysics 6.1 software. An example modeling file is attached in the Supplementary Materials and can be directly run in COMSOL 6.1. In our simulations, w_{PEDOT} varies from 10 to 80 nm while keeping the following parameters fixed $w_{\text{PSS}} = 2w_{\text{PEDOT}}$, $l_p = 5w_{\text{PEDOT}}$, $l_e = 300$ nm, and $v = 1000$ mV s^{-1}. The detailed parameters are listed in Table S1.

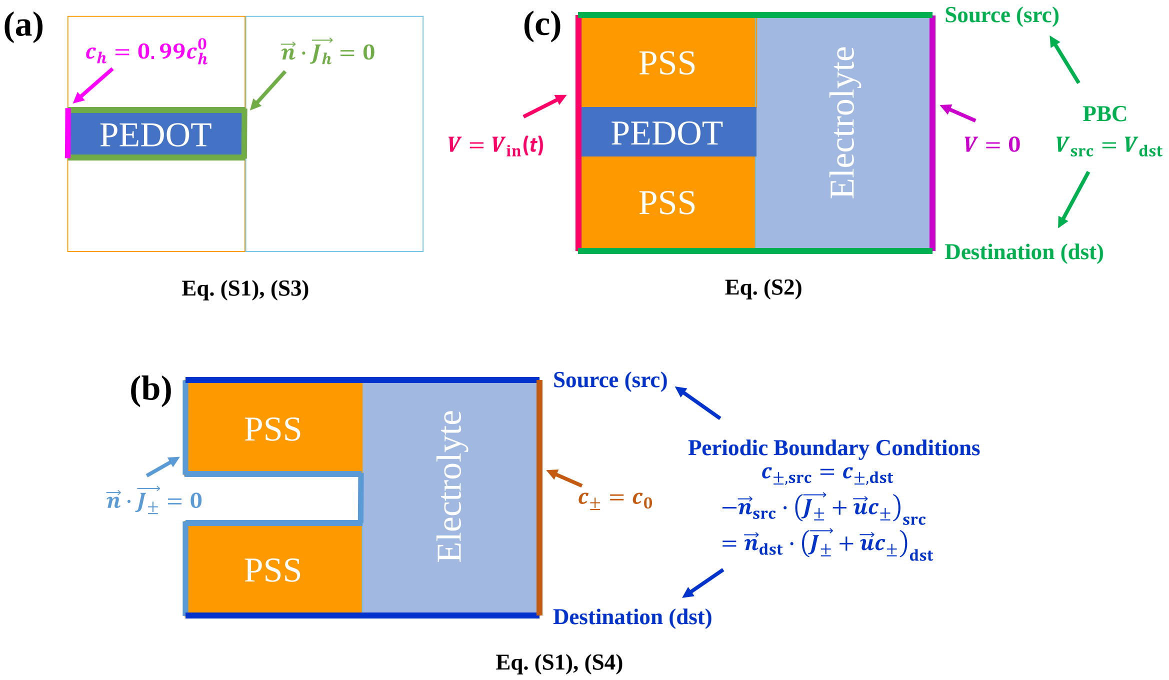

Figure S2: Schematics of the boundary conditions for Eq. (S1)-(S4) at different regions in the 2D two-phase model (half-cell).

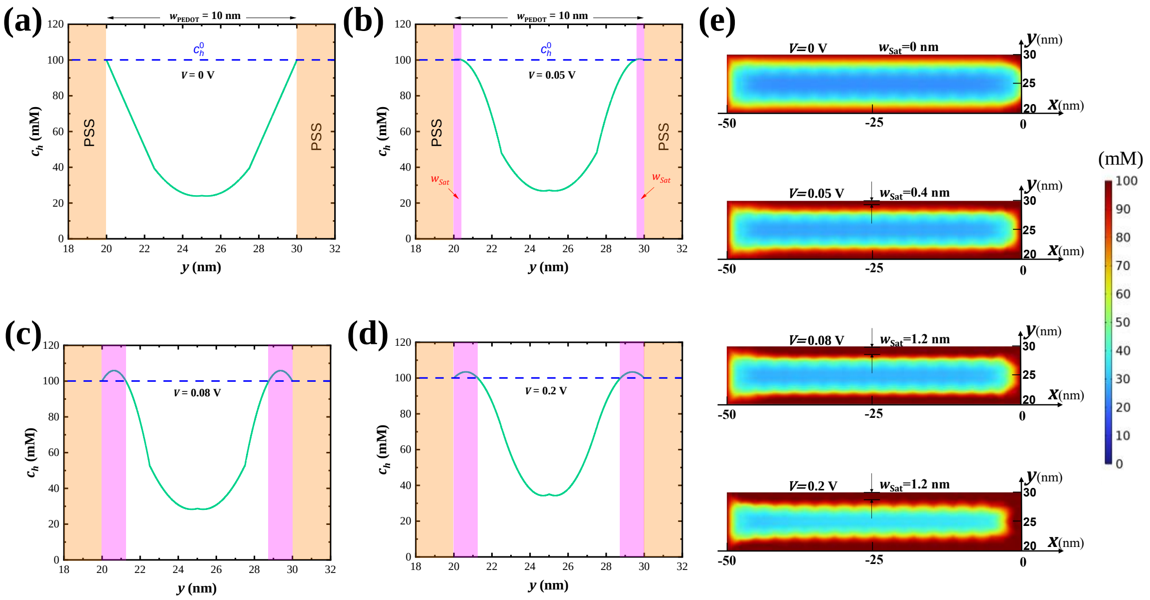

Figure S3: Simulated hole concentration profile. a-d, hole concentration profile at $x = -25$ nm for $w_{PEDOT} = 10$ nm under different applied potential V of (**a**) 0 V, (**b**) 0.05 V, (**c**) 0.08 V and (**d**) 0.2 V. **e**, the corresponding hole concentration distribution in the PEDOT region. From top to bottom, the saturation region width w_{sat} is (**a**) 0 nm, (**b**) 0.4 nm, (**c**) 1.2 nm and (**d**) 1.2 nm. After V increases to a certain value, w_{sat} no longer increases.

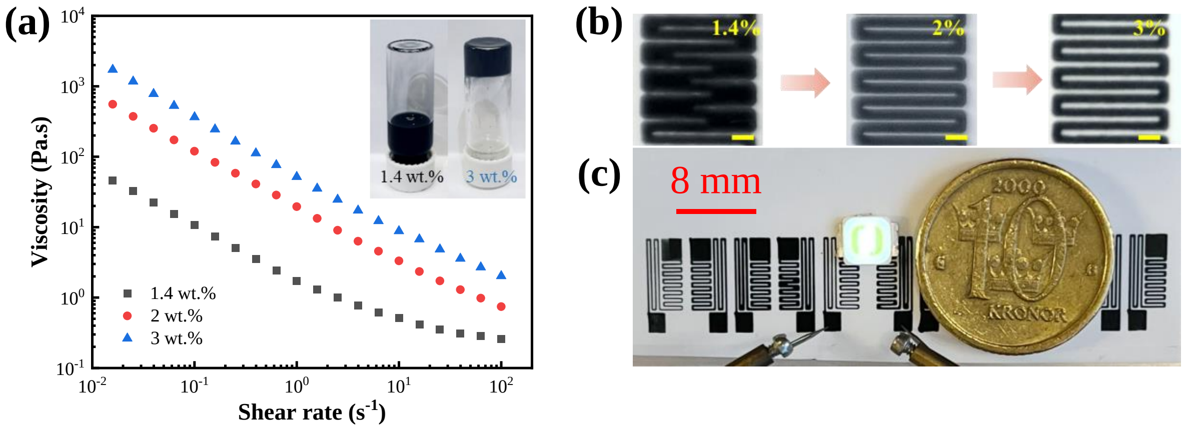

Figure S4: Characterization of PEDOT:PSS DIW inks. a, Viscosity as a function of shear rate for different mass concentration, (inset) Photographs of the 1.4% and 3% inks placed upside down. **b**, Photographs of printed patterns with the inks of different concentration (scale bar 1 mm). **c**, Demo of printed conducting polymer circuits on paper substrates that can be used to light up an LED.

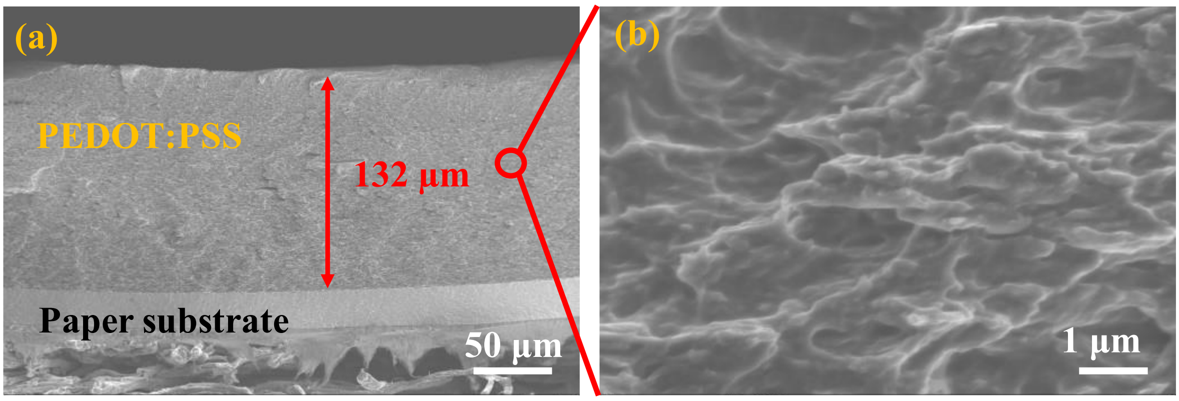

Figure S5: Cross-sectional SEM images of the MSCs with 25 printing passes.

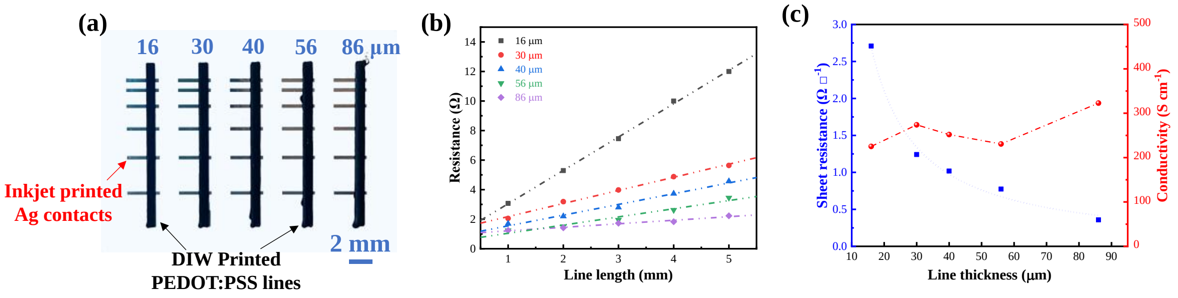

Figure S6: Transmission line method (TLM) used to measure the conductivity of the DIW printed PEDOT:PSS patterns. a, Photograph of the DIW printed PEDOT:PSS lines and inkjet printed silver contacts on photopaper. The PEDOT:PSS lines have length ranging from 1 to 5 mm, and width ranging from 1.2 to 1.5 mm. **b**, Plot of resistance against line length for various thickness (ranging from 16 to 86 µm). **c**, Sheet resistance and conductivity of printed PEDOT:PSS lines.

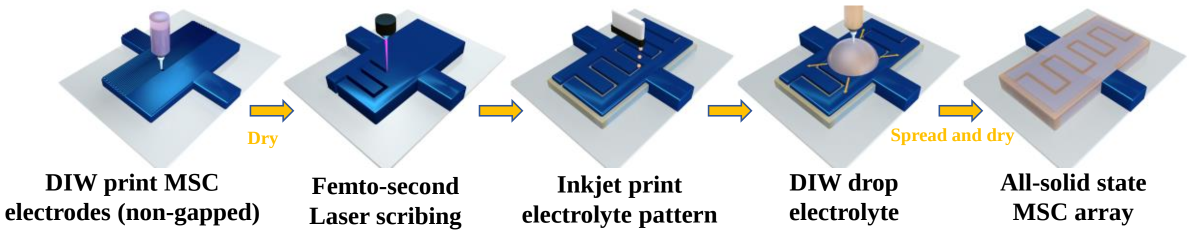

Figure S7: Schematic of the fabrication process of the fully-printed MSC arrays.

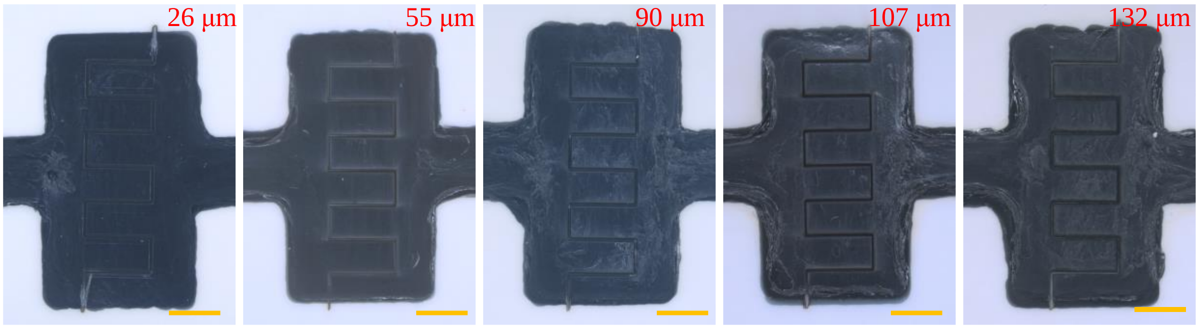

Figure S8: Photographs of the MSC electrodes with different thickness (scale bar: 1mm).

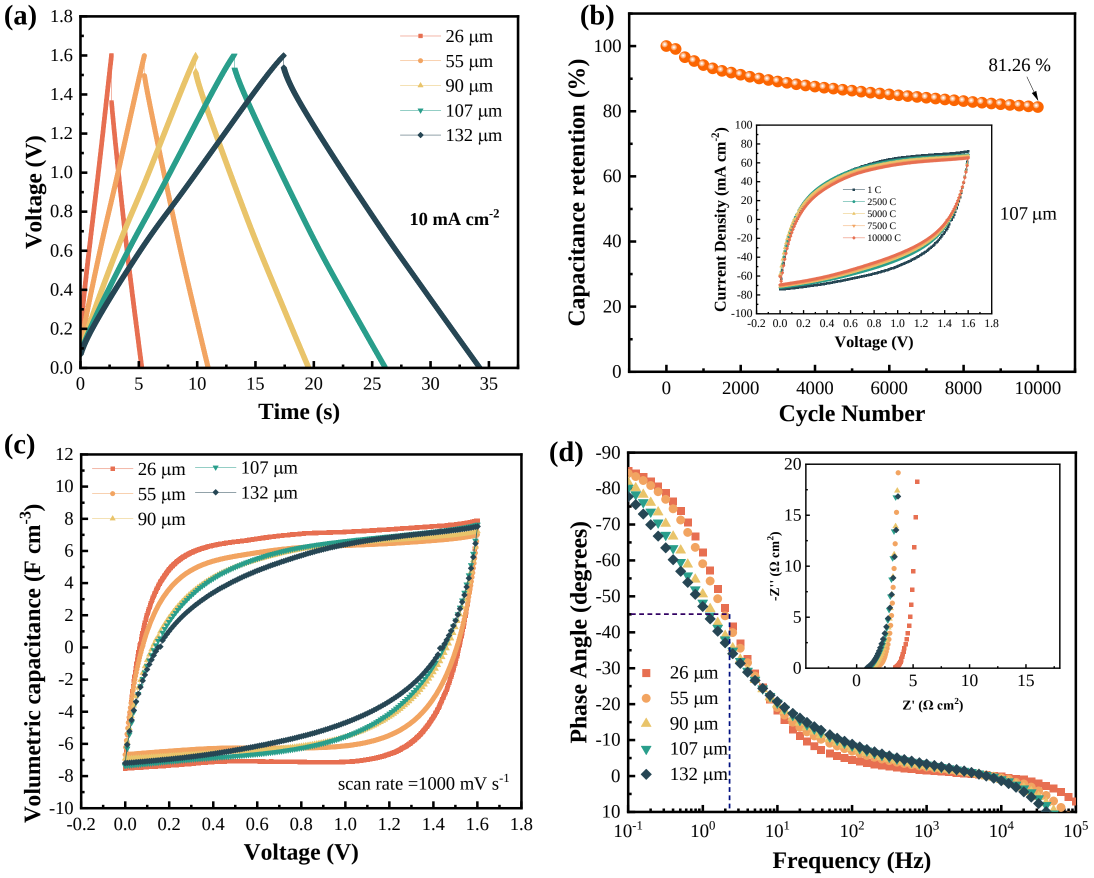

Figure S9: Additional electrochemical characterization of the MSCs with different thickenss. a, GCD curves at current density of 10 mA cm^{-2}. **b**, Cycling performance for 10000 CV tests for the MSC of 107-μm-thick electrode, (inset) CV curves after different number of cycles. **c**, CV curves at the scan rate of 1000 mV s^{-1}. **d**, Bode plot and Nyquist plot (inset) of MSCs.

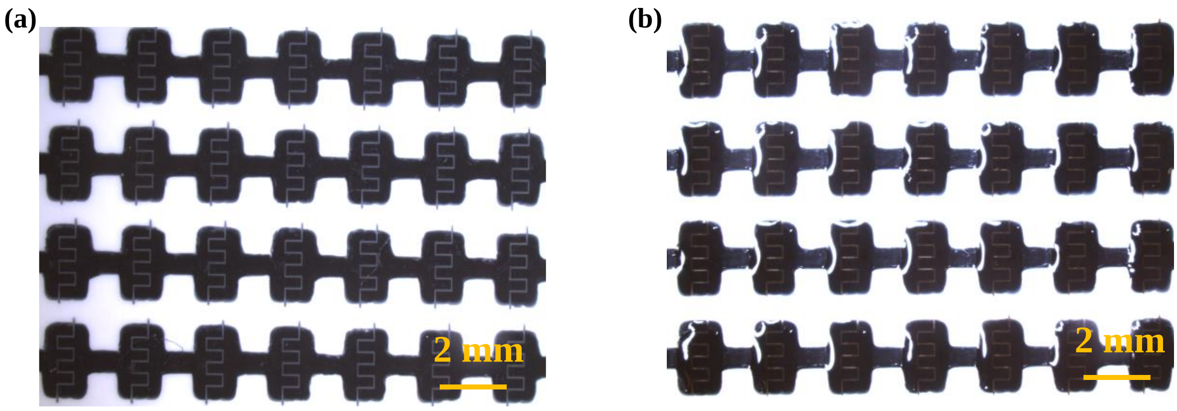

Figure S10: Optical micrographs of one large-scale MSC array. a, Only electrodes (without electrolytes). **b**, Complete MSCs (with electrolytes).

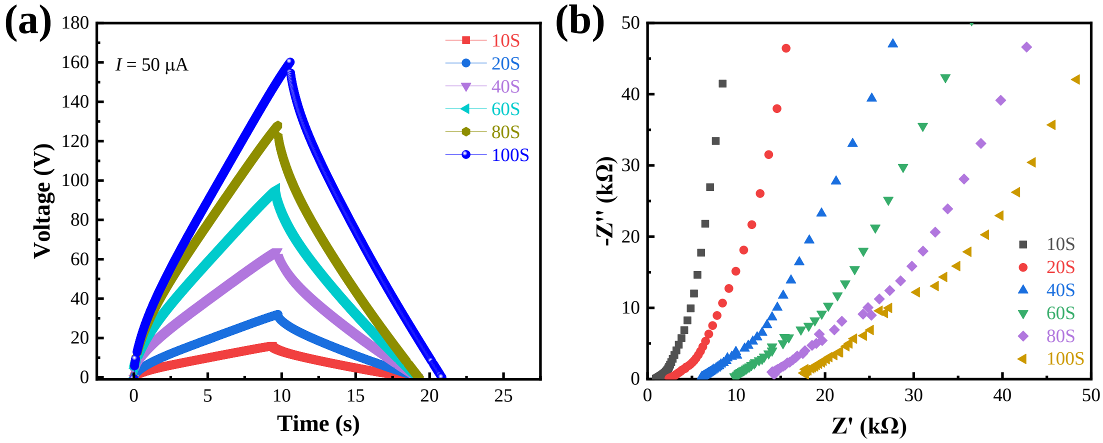

Figure S11: Additional electrochemical performance of the large-scale MSC arrays. a,**b**, GCD curves at 50 µA (**a**) and Nyquist plot (**b**) of the MSC arrays with different cell number.

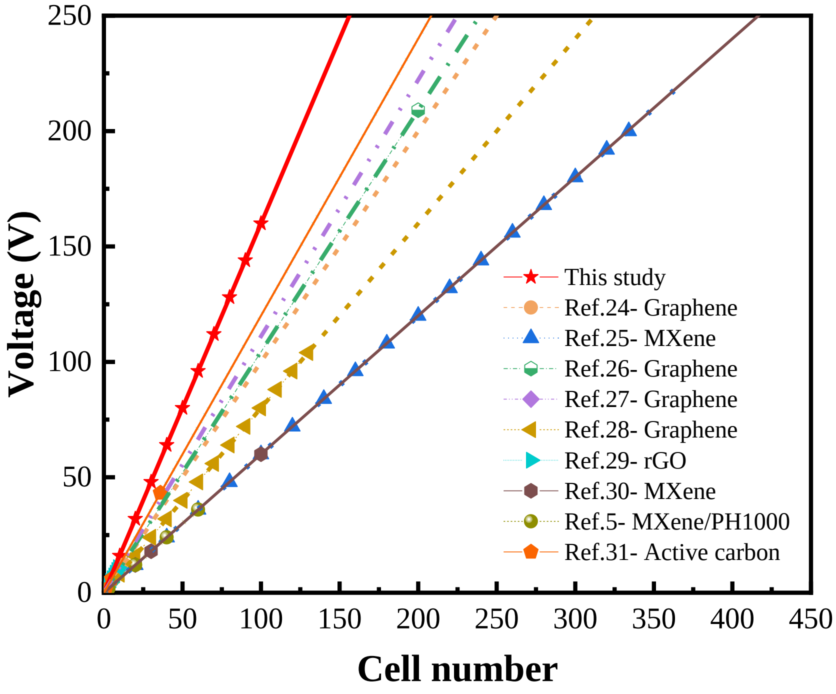

Figure S12: Dependence of WVW on the cell number of the MSC arrays in the literature.

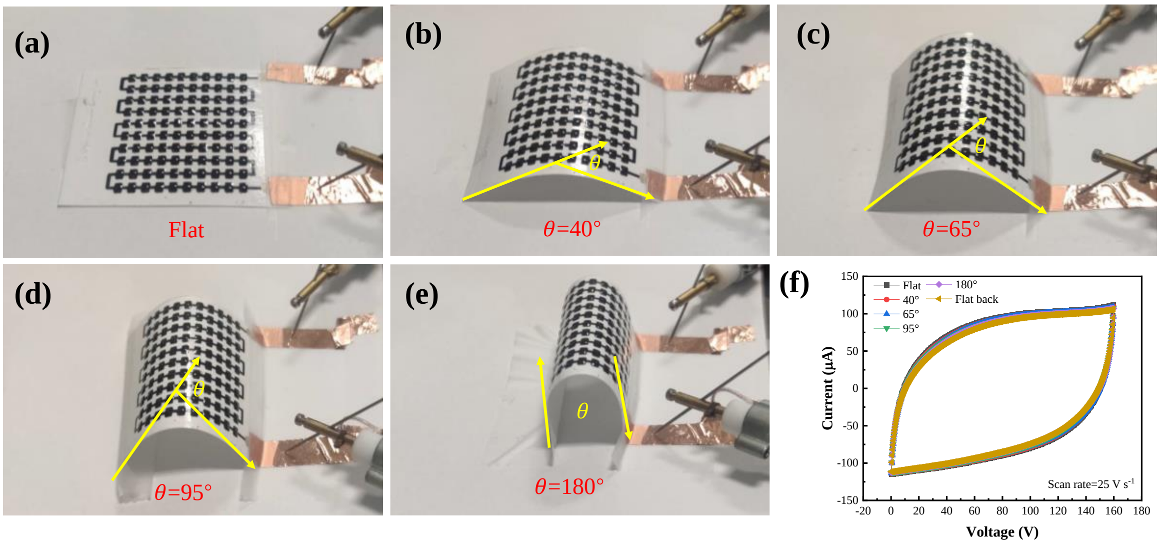

Figure S13: Characterization of flexibility of the large-scale MSC array. Photographs (**a-e**) and CV curves at 25 V s^{-1} (**f**) of the MSC array under the different bending angles.

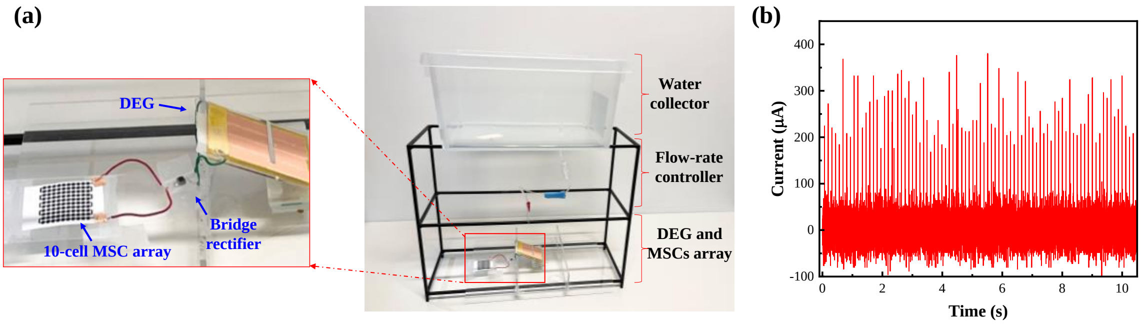

Figure S14: The self-charging power system. a, Photograph of the self-charging power system consisting of the DEG, bridge rectifier and printed 100-cell MSC array. **b**, Output current from DEG with a load resistance of 10 kΩ. The bridge rectifier used in this work is BAS4002A-RPP (Infineon Technologies).

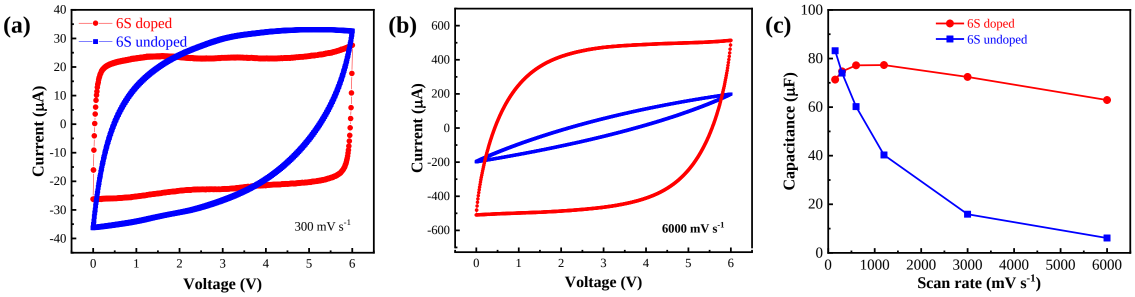

Figure S15: Comparison of rate capability between 6-cell MSC arrays of doped and undoped PEDOT: PSS. a,**b**, CV curves at the scan rate of (**a**) 300 mV s^{-1} and (**b**) 6000 mV s^{-1}. **c**, The overall capacitance versus scan rate.

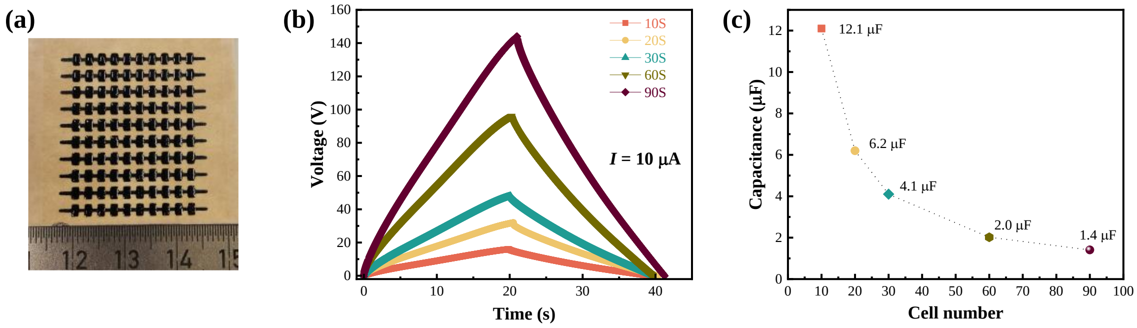

Figure S16: Electrochemical performance of the MSC array of different cell numbers. **a**, Photograph of the MSC array on carton paper substrate (used in Fig. 3d-g, the electrodes are printed with 2 DIW passes of PEDOT:PSS ink). **b**, GCD curves at the current of 10 μA. **c**, Capacitance calculated from the GCD curves.

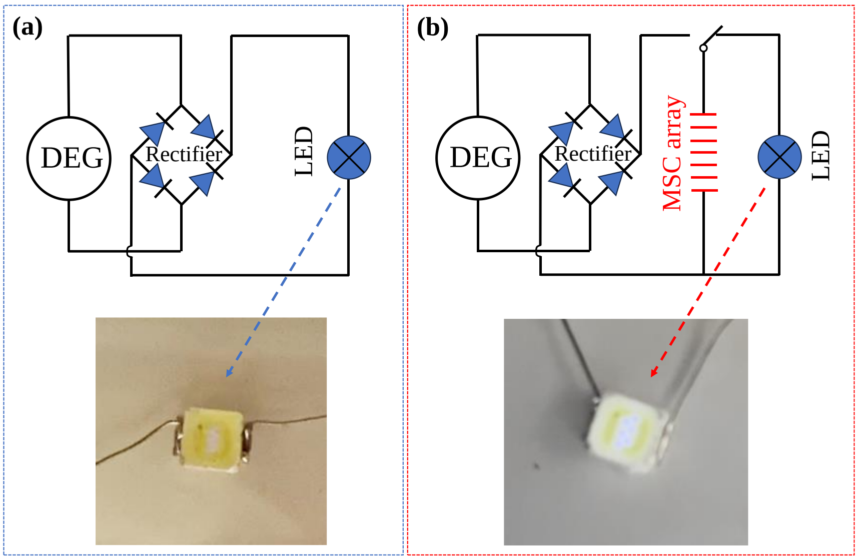

Figure S17: Photographs of an LED powered by the DEG. a, Directly powered by the DEG. **b**, Through a 30-cell MSC array that has been charged by the DEG for 150 s. The upper parts of the panels are the equivalent circuit diagrams.

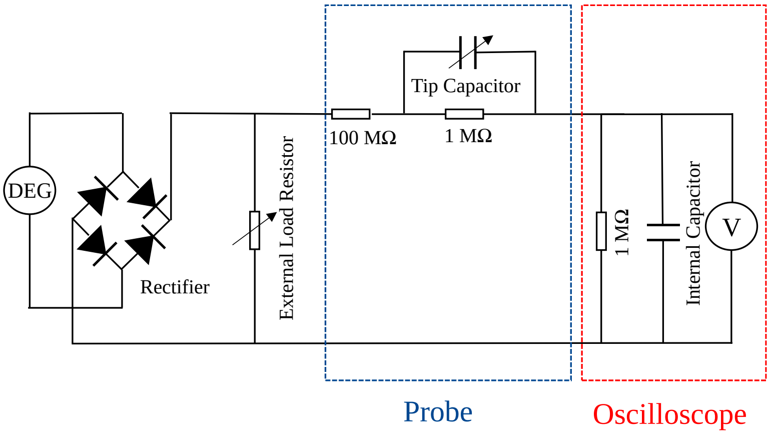

Figure S18: Equivalent circuit diagram for testing average output power of the DEG.

Table S1. The meaning and Initial Value of the parameters in the simulation model.

Parameter	Meaning	Initial Value	Unit
w_{PEDOT}	Width of PEDOT region	10-80	nm
w_{PSS}	Width of PSS region	$2w_{\mathrm{PEDOT}}$	nm
l_p	Length of PEDOT region	$5w_{\mathrm{PEDOT}}$	nm
l_e	Length of electrolyte	300	nm
c_h^0	Saturation hole concentration	100	mol m^{-3}
D_h	Diffusion coefficient of holes	10^{-8}	m^2 s^{-1}
$D_\mp$	Diffusion coefficient of ions in PSS region or electrolyte	10^{-9} (in electrolyte)<br>10^{-14} (in PSS region)	m^2 s^{-1}
c_{max}	Maximum concentration of ions in system	600	mol m^{-3}
c_0	Bulk concentration of electrolyte	10	mol m^{-3}
c_+	Cations concentration in PSS region or electrolyte	0 (in electrolyte)<br>100 (in PSS region)	mol m^{-3}
c_-	Anions concentration in PSS region or electrolyte	0	mol m^{-3}
c_h	Hole concentration in PEDOT region	0	mol m^{-3}
ϵ_r	Relative dielectric permittivity of the system	81	/

Table S2. Summary of the electrochemical performance of the state-of-the-art printed SCs.

Electrode materials	Substate	Metal free	WVW (V)	Electrolyte	CV Capacitance (mF cm^{-2}@ mV s^{-1})	GCD Capacitance (mF cm^{-2}@ mA cm^{-2})	Energy density (μWh cm^{-2})	Power density (mW cm^{-2})	Year & Ref.
PEDOT:PSS	Cellulose/polyester cloth	Yes	0.8	Liquid sweat	10 @ 1	8.45 @ 0.084	1.63	0.40	2020[2]
MnO_2/rGO/ PEDOT:PSS	PET	No	0.8	PVA/CH_3COOLi	12.9 @ 5	12.2 @ 0.17	1.125	1.8	2018[3]
PEDOT:PSS/ MnO_2	3D fabrics	Yes	0.8	PVA/LiCl	/	135.4 @ 0.08	12.03	32	2021[4]
MXene/ PEDOT:PSS	Paper	Yes	0.6	PVA/H_2SO_4	/	22.6 @ 0.015	0.28	4.5	2021[5]
Inkjet printed PDG	Paper	Yes	1	PSSH*/H_3PO_4	3.2 @ 200	4.5 @ 0.025	0.21	1.51	2021[6]
PEDOT:PSS/ CNT/Ag	Paper	No	0.9	PVA/ H_3PO_4	0.43@ 10	/	0.0422	0.089	2016[7]
PEDOT/ cellulose	Cellulose paper	Yes	1.2	PVA/H_2SO_4	/	115 @ 0.4	17	0.884	2015[8]
PEDOT/Au	PEN	No	0.8<br>1.5	PVA/H_2SO_4<br>[EMIM][TFSI]/ PVDF	3.7 @ 2000	9 @ 0.035	1.85	0.042	2015[9]
PEDOT hydrogel	Freestanding	Yes	0.8	PVA/H_2SO_4	/	177 @ 1	15.73	0.4	2022[10]
CNT/PANI	Silicone rubber	Yes	0.8	PVA/H_3PO_4	/	167 @ 0.4	14.9	0.29	2019[11]
Clay-like MXene	Paper	No	0.6	H_2SO_4	25 @ 20	/	0.77	46.6	2016[12]
Graphite/activated carbon	CNF/ CNC /glycerol substrate	Yes	1.2	CNC/NaCl	25 @ 1	/	0.84	0.797	2021[13]
PEDOT:PSS/ CNF	Aluminum foil	No	0.6	HEC/EMIM:ES	/	9.1 @ 0.05	0.453	26.5	2022[14]
MXene	Paper	Yes	0.5	PVA/H_3PO_4	/	43 @ 0.005	0.32	0.0114	2019[15]
MXene sediment	Paper	Yes	0.6	PVA/H_3PO_4	134 @ 2	158 @ 0.08	1.64	0.78	2020[16]
MnHCF-MnO_x/ErGO	PET	No	1	PVA/LiCl	/	16.8 @ 0.1	2.3	0.5	2020[17]
Ni/MnO_2	Kapton	No	1	CMC/Na_2SO_4	4.15 @ 20	/	0.18	0.42	2018[18]

PG/WJM-graphene:SWCNTs	PET	Yes	1.8	PVA/H_3PO_4	/	1.324 @ 0.0125	0.064	20	2019[19]
3D graphene-cellulose	Paper	Yes	1	PVA/H_2SO_4	10.1 @ 5	9.8 @ 0.1	1.35	0.54	2022[20]
MXene aerogels	Silicon wafer	No	0.6	PVA/H_2SO_4	79 @ 10	/	2	0.6	2021[21]
MXene/AgNW/MONW	PDMS	No	0.8	PVA/KOH	216.2 @ 10	/	6.47	58.3	2020[22]
Activated carbon	PET	Yes	3	[EMIM][TFSI] liquid	/	171.7 @ 1.6	103.4	2.53	2021[23]
PEDOT–26 µm	Paper	Yes	1.6	PSSH/H_3PO_4	16 @ 500	20.15 @ 0.8	4.76	21.4	This work
PEDOT–55 µm					30 @ 400	38.37 @ 0.8	8.47	38.0	
PEDOT–90 µm					51.4 @ 300	68.21 @ 0.8	11.56	52.0	
PEDOT-107 µm					65.5 @ 200	91.22 @ 0.8	13.63	61.3	
PEDOT–132 µm					80 @ 200	116.59 @ 0.8	15.16	68.3	

Table S3. Summary of the electrochemical performance of the state-of-the-art MSC arrays.

Electrode	Manufacturing method	Cell number	Highest WVW(V)	Overall capacitance (μF)	Ref.
Graphene	Inkjet printing	144	12	94	[24]
MXene	Lithography/spray coating	334	200	0.02	[25]
Graphene	Laser scribing	200	209	0.43	[26]
Graphene	Laser scribing	9	10	124	[27]
Graphene	Screen printing	130	104	1.3	[28]
ErGO	Electrodeposition/laser scribing	9	10.8	58.8	[29]
MXene	Screen printing	100	60	/	[30]
MXene/PH1000	Inkjet printing	60	36	/	[5]
Active carbon	Electrohydrodynamic jet printing	36	43.2	/	[31]
PEDOT:PSS	DIW/Femtosecond laser/ Inkjet printing	10	16	30	This work
PEDOT:PSS	DIW/Femtosecond laser/ Inkjet printing	100	160	3	This work

Table S4. Summary of the energy storage performance of the state-of-the art self-charging power systems.

Energy Harvester	Energy Storage Device	Power Management Circuit	Peak Voltage of Harvester (V)	ESE (%)	Ref.
PTFE-graphene TENG	Zn-Ion Batteries	rectifier	15	39.8	[32]
PTFE-Cu TENG	Sodium Batteries	transformer and rectifier	6.6	54	[33]
Radial-arrayed rotary TENG	Sodium-Ion Batteries	rectifier	12	62.3	[34]
Rotating RF Pulsed TENG	10 μF capacitor	inductor, diode, capacitor<br>capacitor	720	52.0<br>0.04	[35]
Perovskite Solar Cell	Li-Ion Batteries	none	3.8	60	[36]
Radial-arrayed rotary TENG	Li-Ion Batteries	transformer and rectifier	4.3	66.1<br>73.6<br>74.4<br>83.4	[37]
PTFE-Cu TENG	Li-Ion Batteries	transformer and rectifier	9.2<br>15.4<br>61.9<br>391.4	72.4<br>40.1<br>8.4<br>1.2	[38]
DEG	MSC arrays	rectifier	150	62	This work

References

[1] A. V. Volkov, K. Wijeratne, E. Mitraka, U. Ail, D. Zhao, K. Tybrandt, J. W. Andreasen, M. Berggren, X. Crispin, I. V. Zozoulenko, *Adv Funct Mater* **2017**, 27, 1700329.

[2] L. Manjakkal, A. Pullanchiyodan, N. Yogeswaran, E. S. Hosseini, R. Dahiya, *Adv Mater* **2020**, 32, 1907254.

[3] Y. Lin, J. Chen, M. M. Tavakoli, Y. Gao, Y. Zhu, D. Zhang, M. Kam, Z. He, Z. Fan, *Adv Mater* **2019**, 31, 1804285.

[4] D. Li, S. Yang, X. Chen, W. Y. Lai, W. Huang, *Adv Funct Mater* **2021**, 31, 2107484.

[5] J. Ma, S. Zheng, Y. Cao, Y. Zhu, P. Das, H. Wang, Y. Liu, J. Wang, L. Chi, S. Liu, Z. S. Wu, *Adv Energy Mater* **2021**, 11, 2100746.

[6] Z. Li, V. Ruiz, V. Mishukova, Q. Wan, H. Liu, H. Xue, Y. Gao, G. Cao, Y. Li, X. Zhuang, J. Weissenrieder, S. Cheng, J. Li, *Adv Funct Mater* **2021**, 32, 2108773.

[7] W. Liu, C. Lu, H. Li, R. Y. Tay, L. Sun, X. Wang, W. L. Chow, X. Wang, B. K. Tay, Z. Chen, J. Yan, K. Feng, G. Lui, R. Tjandra, L. Rasenthiram, G. Chiu, A. Yu, *Journal of Materials Chemistry A* **2016**, 4, 3754.

[8] B. Anothumakkool, R. Soni, S. N. Bhange, S. Kurungot, *Energ Environ Sci* **2015**, 8, 1339.

[9] N. Kurra, M. K. Hota, H. N. Alshareef, *Nano Energy* **2015**, 13, 500.

[10] T. Cheng, F. Wang, Y.-Z. Zhang, L. Li, S.-Y. Gao, X.-L. Yang, S. Wang, P.-F. Chen, W.-Y. Lai, *Chemical Engineering Journal* **2022**, 450.

[11] V. Rajendran, A. M. V. Mohan, M. Jayaraman, T. Nakagawa, *Nano Energy* **2019**, 65, 104055.

[12] N. Kurra, B. Ahmed, Y. Gogotsi, H. N. Alshareef, *Adv Energy Mater* **2016**, 6, 1601372.

[13] X. Aeby, A. Poulin, G. Siqueira, M. K. Hausmann, G. Nystrom, *Adv Mater* **2021**, 33, 2101328.

[14] M. G. Say, C. J. Brett, J. Edberg, S. V. Roth, L. D. Soderberg, I. Engquist, M. Berggren, *ACS Appl Mater Interfaces* **2022**, 14, 55850.

[15] C. Zhang, L. McKeon, M. P. Kremer, S.-H. Park, O. Ronan, A. Seral-Ascaso, S. Barwich, C. Ó. Coileáin, N. McEvoy, H. C. Nerl, B. Anasori, J. N. Coleman, Y. Gogotsi, V. Nicolosi, *Nature Communications* **2019**, 10.

[16] S. Abdolhosseinzadeh, R. Schneider, A. Verma, J. Heier, F. Nuesch, C. J. Zhang, *Adv Mater* **2020**, 32, 2000716.

[17] J. Liang, B. Tian, S. Li, C. Jiang, W. Wu, *Adv Energy Mater* **2020**, 10, 2000022.

[18] Y. Chen, X. Li, Z. Bi, G. Li, X. He, X. Gao, *Chemical Engineering Journal* **2018**, 353, 499.

[19] S. Bellani, E. Petroni, A. E. Del Rio Castillo, N. Curreli, B. Martín-García, R. Oropesa-Nuñez, M. Prato, F. Bonaccorso, *Advanced Functional Materials* **2019**, 29.

[20] X. Jiang, R. Gao, G. Liu, H. Luo, X. Zhao, L. Jiang, *Small Methods* **2022**, 6, e2101454.

[21] H. Tetik, J. Orangi, G. Yang, K. Zhao, S. B. Mujib, G. Singh, M. Beidaghi, D. Lin, *Adv Mater* **2022**, 34, e2104980.

[22] X. Li, H. Li, X. Fan, X. Shi, J. Liang, *Adv Energy Mater* **2020**, 10, 1903794.

[23] C. Gao, J. Huang, Y. Xiao, G. Zhang, C. Dai, Z. Li, Y. Zhao, L. Jiang, L. Qu, *Nat Commun* **2021**, 12, 2647.

[24] J. Li, S. Sollami Delekta, P. Zhang, S. Yang, M. R. Lohe, X. Zhuang, X. Feng, M. Ostling, *ACS Nano* **2017**, 11, 8249.
[25] S. Wang, L. Li, S. Zheng, P. Das, X. Shi, J. Ma, Y. Liu, Y. Zhu, Y. Lu, Z.-S. Wu, H.-M. Cheng, *Natl Sci Rev* **2022**, nwac271.
[26] X. Li, W. Cai, K. S. Teh, M. Qi, X. Zang, X. Ding, Y. Cui, Y. Xie, Y. Wu, H. Ma, Z. Zhou, Q. A. Huang, J. Ye, L. Lin, *ACS Appl Mater Interfaces* **2018**, 10, 26357.
[27] S. Bai, Y. Tang, Y. Wu, J. Liu, H. Liu, W. Yuan, L. Lu, W. Mai, H. Li, Y. Xie, *ACS Appl Mater Interfaces* **2020**, 12, 45541.
[28] X. Shi, S. Pei, F. Zhou, W. Ren, H.-M. Cheng, Z.-S. Wu, X. Bao, *Energ Environ Sci* **2019**, 12, 1534.
[29] N. Kamboj, T. Purkait, M. Das, S. Sarkar, K. S. Hazra, R. S. Dey, *Energy & Environmental Science* **2019**, 12, 2507.
[30] S. Zheng, H. Wang, P. Das, Y. Zhang, Y. Cao, J. Ma, S. F. Liu, Z. S. Wu, *Adv Mater* **2021**, 33, 2005449.
[31] K.-H. Lee., S.-S. Lee., D. B. Ahn., J. Lee., D. Byun., S.-Y. Lee., *Science Advances* **2020**, 6, eaaz1692.
[32] Z. Wang, Z. Ruan, W. S. Ng, H. Li, Z. Tang, Z. Liu, Y. Wang, H. Hu, C. Zhi, *Small Methods* **2018**, 2, 1800150.
[33] Y. Lu, L. Lu, G. Qiu, C. Sun, *ACS Appl Mater Interfaces* **2020**, 12, 39342.
[34] H. Hou, Q. Xu, Y. Pang, L. Li, J. Wang, C. Zhang, C. Sun, *Adv Sci* **2017**, 4, 1700072.
[35] W. Shang, G. Gu, W. Zhang, H. Luo, T. Wang, B. Zhang, J. Guo, P. Cui, F. Yang, G. Cheng, Z. Du, *Nano Energy* **2021**, 82, 105725.
[36] J. Xu, Y. Chen, L. Dai, *Nat Commun* **2015**, 6, 8103.
[37] X. Nan, C. Zhang, C. Liu, M. Liu, Z. L. Wang, G. Cao, *ACS Appl Mater Interfaces* **2016**, 8, 862.
[38] X. Pu, M. Liu, L. Li, C. Zhang, Y. Pang, C. Jiang, L. Shao, W. Hu, Z. L. Wang, *Adv. Sci.* **2016**, 3, 1500255.
